# Protective effects of miR-24-2-5p in early stages of breast cancer bone metastasis

**DOI:** 10.1186/s13058-024-01934-2

**Published:** 2024-12-18

**Authors:** Margherita Puppo, Martine Croset, Davide Ceresa, Manoj Kumar Valluru, Victor Gabriel Canuas Landero, Monserrat Hernandez Guadarrama, Michele Iuliani, Francesco Pantano, Penelope Dawn Ottewell, Philippe Clézardin

**Affiliations:** 1 Research Unit UMR_S1033, LyOS, Faculty of Medicine Lyon-Est, INSERM, 7 Rue Guillaume Paradin, Lyon, 69372 France; 2 Université Claude Bernard Lyon 1, Villeurbanne, France; 3 Division of Clinical Medicine, School of Medicine and Population Health, University of Sheffield, Sheffield, UK; 4 IRCCS Ospedale Policlinico San Martino, Genova, Italy; 5 Medical Oncology, Fondazione Policlinico Universitario Campus Bio-Medico, Via Alvaro del Portillo, Roma, 200 - 00128 Italy; 6 Department of Medicine and Surgery, Università Campus Bio-Medico di Roma, Via Alvaro del Portillo, Roma, 21 - 00128 Italy

**Keywords:** Breast cancer, Bone metastasis, Early-stage, Osteoclasts, miR-24, Small extracellular vesicles, Migration, Invasion, Proliferation, Circulating miRNAs

## Abstract

**Background:**

Bone is the most frequent site of metastasis for breast cancer (BC). Metastatic BC cells interact with bone cells, including osteoclasts and osteoblasts, creating a cancer niche where they seed and proliferate. MicroRNAs (miRNAs) are regulators of breast-to-bone metastasis progression. MiR-24-2-5p has previously been shown to have roles in both breast cancer progression and inhibition of osteogenic differentiation. However, a direct link between miR-24-2-5p activity and the onset of bone metastasis remains ill-defined.

**Methods:**

Analysis of the expression of miR-24 forms (miR-24-2-5p, miR-24-3p, miR-24-1-5p) in the serum from early-stage BC patients at baseline (time of surgery) was conducted. MiR-24-2-5p overexpression in BC cells (NW1, a *luc2*-positive subpopulation of MDA-MB-231, and MCF7) was obtained by miRNA mimic transfection or lentivirus transduction. MiR-24-2-5p downregulation in BC cells (ZR-75-1, T-47D, SK-BR-3) was obtained by miRNA inhibitor transfection. Cell proliferation, migration and/or invasion assays were performed to assess BC cell functions after modulation of miR-24-2-5p expression. An animal model was used to assess the effect of miR-24-2-5p overexpression on early BC metastasis formation, as judged by bioluminescence imaging, and on bone remodelling, following measurement of circulating bone resorption (CTX-I) and bone formation (P1NP) markers. The effect of conditioned medium from miR-24-2-5p-overexpressing BC cells on human and murine osteoclast differentiation was investigated. Endogenous miR-24-2-5p expression levels were also quantified during murine osteoclast differentiation. RNA-sequencing (RNA-seq) analysis of BC cells was performed to evaluate transcriptomic changes associated with miR-24-2-5p overexpression. Selected modulated transcripts upon miR-24-2-5p overexpression were further validated by real-time qPCR.

**Results:**

Low expression levels of miR-24-2-5p, but not other miR-24 forms (miR-24-3p, miR-24-1-5p), in the serum from early-stage BC patients were associated with a high risk to develop future (bone) metastases. MiR-24-2-5p was also present in small extracellular vesicles secreted from BC cells. Forced expression of miR-24-2-5p in BC cells (NW1, MCF7) reduced their malignant traits (migration, invasion, and proliferation) in vitro. Furthermore, miR-24-2-5p overexpression in NW1 cells reduced metastasis, particularly in bone, and decreased bone turnover in vivo. RNA-seq and real-time qPCR analyses of NW1 and MCF7 cells overexpressing miR-24-2-5p showed the downregulation of common transcripts (CNNM4, DCTD, FMR1, PIGS, HLA-A, ICK, SH3BGRL2, WDFY, TRAF9B, IL6ST, PEX10, TRIM59). The conditioned medium from BC cells overexpressing miR-24-2-5p decreased human and murine osteoclast differentiation in vitro. Additionally, endogenous miR-24-2-5p expression levels in murine bone marrow-derived monocytes decreased during their differentiation into osteoclasts, further suggesting an inhibitory role for miR-24-2-5p during osteoclastogenesis.

**Conclusion:**

MiR-24-2-5p exerts multiple protective roles in the early steps of BC bone metastasis by reducing malignant BC cell traits and tumour cell dissemination in bone, as well as by reducing the differentiation of precursors into mature osteoclasts.

**Supplementary Information:**

The online version contains supplementary material available at 10.1186/s13058-024-01934-2.

## Introduction

Breast cancer (BC) metastasis remains a major challenge in the clinic, being the leading cause of death in BC patients worldwide [1]. BC metastasis occurs when tumour cells that reside in the breast break away from the primary site and disseminate through the bloodstream or lymphatic system to other parts of the body. Bone is the most common site of BC metastasis [2]. The 5-year overall survival rate of patients with early BC is between 74 and 89% in European countries [3], thanks to the existence of a very efficient armamentarium of screening tests (i.e., mammography, ultrasounds, magnetic resonance imaging and physical examination) and therapies (surgical resection, radio- and chemo-therapy, hormone therapy, and/or targeted therapy) [4]. However, when BC tumour cells spread to distant organs, the disease is considered to be incurable, and the median overall survival for metastatic patients is between 2 and 3 years [5, 6]. In the case of BC bone metastasis, current treatments aim to palliate morbidity associated with skeletal lesions [2]. These include systemic treatments blocking osteoclast-mediated bone destruction (bisphosphonate or denosumab) and local treatments (e.g., targeted radionucleotide therapy, cementoplasty and/or orthopedic surgery when pathological fractures occur) [2]. Thus, prevention of bone metastases is a key objective and, to achieve this, it is crucial to better understand molecular mechanisms that control cellular events that precede the development of overt skeletal lesions.

MicroRNAs (miRNAs) are a class of small non-coding RNA molecules that post-transcriptionally regulate gene expression in cells [13]. Specifically, miRNAs play a central role in the regulation of cell differentiation, proliferation, and survival by binding to complementary target messenger RNAs (mRNAs), resulting in mRNA translational inhibition or degradation [13]. More than 60% of human protein-coding genes contain at least one conserved miRNA-binding site, and most protein-coding genes are predicted to be under the control of miRNAs [7]. MiRNAs are found to be dysregulated in numerous diseases, including cancer [8]. In BC, the dysregulation of miRNA expression has been implicated from the development of the primary tumour to the formation of metastases in distant organs, such as bone [8, 9]. We previously showed that high levels of miR-662 in serum at baseline (time of surgery) from patients with early BC are associated with future recurrence in distant organs, and that overexpression of miR-662 in BC cells promotes metastatic dissemination in animal models [10]. Specifically, the overexpression of miR-662 was sufficient to increase proliferative, migratory, and stem-like properties of BC cells, allowing them to successfully colonise distant organs [10]. In this previous study, we also observed that other miRNAs, such as miR-24-2-5p, were associated with (bone) relapse in BC [10].

MiR-24-2-5p belongs to the *miR-23* ∼ *27* ∼ *24* clusters, whose members have been extensively investigated for their role in several diseases, including cancer [11]. In the context of BC, miR-24-2 precursor (pre-miR-24-2) decreases BC tumorigenesis, with the miR-24-2-5p mature form being responsible for repressing PKC-alpha levels, which is important for BC cell survival [12]. In bone, miR-24-2-5p has been shown to inhibit osteogenic differentiation [13–15]. A role for miR-24-2-5p in BC (bone) metastasis formation has however never been explored. In the present study, we identified circulating miR-24-2-5p as the only form of miR-24 to be associated to (bone) recurrence in early-stage BC patients. Moreover, we unrevealed that miR-24-2-5p overexpression in human BC cells with high metastatic potential reduced tumour progression by exerting a dual inhibitory role (1) on BC cells —reducing their proliferative, migratory, and invasive cell properties in vitro, and bone metastasis in vivo— and (2) on bone-resorbing osteoclasts —inhibiting their differentiation in vitro and bone resorption in vivo.

## Materials and methods

### MiRome analysis on patients’ serum samples

The expression levels for miR-24 forms in serum samples from 48 early-stage BC patients were obtained from our published dataset [10], where a miRome screen was conducted by TaqMan low-density arrays (TLDA). In this dataset, patients have been divided into 3 groups based on clinical information on patients’ metastatic status: NOMET (*n* = 16) with no metastatic recurrence, BONEMET (*n* = 16) with a first relapse in bone, and SOFTMET (*n* = 16) with a first relapse in other tissues than bone [10]. Clinical details on BC patients’ cohort can be found in supplementary Fig. S1. Raw cycle threshold (CT) values, normalized delta CTs (ΔCT) (by global mean normalization), and expression levels for miR-24-1-5p, miR-24-2-5p, miR-24-3p were retrieved from this dataset as well as data on the differential expression of these miRNAs between NOMET and BONEMET groups.

### Receiver operating characteristic (ROC) curves analysis

Combined ROC curve analysis was constructed to evaluate the value of circulating miR-24-2-5p levels combined with tumour grade patients’ information in distinguishing between NOMET and BONEMET groups. Areas under the ROC curves (AUC) were calculated based on 2^−ΔCT^ values.

### Cell lines and cell culture

MDA-MB-231, T-47D, MCF7, BT-474, ZR-75-1, SK-BR-3, and Hs-578T human breast cancer lines were obtained from the American Type Culture Collection (ATCC) and authenticated in-house by DNA fingerprinting, using short tandem repeat method of 10 loci. MDA-MB-231-l*uc2*-NW1 (NW1) cell line was obtained from Dr Ning Wang, University of Sheffield, Sheffield, UK [16]. NW1/LENTI-Ctrl-GFP + were generated by lentivirus transduction as previously described [10]. MDA-MB-231, NW1, T-47D, MCF7, Hs-578T, and BT-474 cell lines were maintained in Dulbecco’s modified Eagle’s medium (DMEM) containing 4.5 g/L glucose (GlutaMAX, Gibco, UK), ZR-75-1 in RPMI 1640 medium (Gibco, UK), SK-BR-3 in McCoy’s 5 A medium (Sigma), supplemented with 10% (v/v) FCS (Invitrogen) and 100 U/mL penicillin/streptomycin, at 37 °C, 5% CO_2_. Cell cultures were routinely tested for mycoplasma contamination (MycoAlert PLUS Mycoplasma Detection Kit, Lonza). Cell lines in culture were used with a maximum of 20 passages after receipt.

### MiR-24-2-5p transient and stable overexpression in BC cells

Transient miR-24-2-5p overexpression in BC cell lines (NW1/MIMIC-miR-24-2-5p, MCF7/MIMIC-miR-24-2-5p) was obtained by cell transfection with Lipofectamine2000 (Invitrogen) using 50 nM of miR-24-2-5p mimics (GenePharma, Shanghai). Control cells (NW1/MIMIC-negCTRL, MCF7/MIMIC-negCTRL) were transfected with 50 nM FAM-labelled negative control mimics (GenePharma, Shanghai). Transfected cells were used for in vitro and in vivo experiments at least 24 h after transfection.

Stable miR-24-2-5p cell lines (NW1/miR-24-5p, MCF/miR-24-5p) were generated by lentivirus transduction with a shMIMIC human lentiviral microRNA hsa-miR-24-2-5p hCMV-TurboGFP (Horizon Discovery, United Kingdom) at 10 MOI, and clones were selected for 2 weeks with 8 µg/mL puromycin. Control cell line for NW1 and MCF7 were obtained by transduction of SMARTvector non-targeting hCMV-TurboGFP control particles as previously described [10].

### MiR-24-2-5p transient downregulation in BC cells

Transient miR-24-2-5p downregulation in BC cell lines (ZR-75-1/INHIB-miR-24-2-5p, T-47D/INHIB-miR-24-2-5p, SK-BR-3/INHIB-miR-24-2-5p) was obtained by cell transfection with Lipofectamine2000 (Invitrogen) using 50 nM of hsa-miR-24-2-5p mirVana miRNA inhibitor (Ambion, Thermo Fisher). Control cells (ZR-75-1/INHIB-negCTRL, T-47D/INHIB-negCTRL, SK-BR-3/INHIB-negCTRL) were transfected with 50 nM mirVana miRNA inhibitor Negative Control #1 (Ambion, Thermo Fisher). Transfected cells were used for in vitro experiments at least 24 h after transfection.

### MiR-24-2-5p quantification in BC cells

Mir-24-2-5p expression levels were quantified by TaqMan real-time quantitative PCR (RT-qPCR) following manufacturers’ instructions. Total RNA was extracted with miRNeasy kit (Qiagen), following manufacturers’ instructions. RNA concentration and purity were evaluated using a Nanodrop™ 2000 spectrophotometer (Thermo Scientific). A reverse transcription into cDNA was performed with TaqMan microRNA RT kit and specific probes directed to hsa-U6 and hsa-miR-24-2-5p (TaqMan miRNA Assays). Real-time qPCR was performed with TaqMan Universal Master Mix NO UNG. Relative gene expression was calculated using the 2^−ΔΔCT^ method.

### Quantification of transcript expression levels in BC cells and osteoclasts

RNA from cell pellet was extracted as described above. Reverse transcriptions into cDNA were performed with iScript cDNA Synthesis Kit (Bio-Rad), and real-time qPCR was carried out with SsoAdvance Universal SYBR Green Supermix (Bio-Rad), following manufacturers’ instructions. Relative gene expression was calculated using the 2^−ΔΔCT^ method. Primer sequences are listed in supplementary Table S1 or were previously published [17].

### Small extracellular vesicle (sEV) isolation by ultracentrifugation

NW1 cells were seeded at a low percentage of confluency (20–30%) and cultured in complete medium for 24 h. The complete medium was then replaced with sEV-free medium (sEV-deprived medium by ultra-centrifugation). After 72 h in culture, the conditioned medium was collected, and sequential centrifugations at 4 °C were performed (300*g for 10 min, 10.000*g for 10 min, 100.000*g for 70 min). An Optima XPN-80 Beckman Coulter Ultracentrifuge was used for the ultra-centrifugation steps. sEV pellet was resuspended in QIAzol (Qiagen) for downstream RNA analysis.

### Proliferation assay

Twenty-four hours post-transfection, cells were seeded into 12-well plates (Costar), in triplicate, at a concentration of 1 × 10^4^ cells/500 µL/well. For 5 consecutive days (day 0, 1, 2, 3, 4), cells were washed with PBS, fixed with 4% (v/v) paraformaldehyde (Fisher Scientific, UK), and stained with crystal violet solution (Sigma-Aldrich). The optical density (590 nm) of a solubilized crystal violet solution in 10% (v/v) acetic acid was measured using a SpectraMax M5e microplate reader (Molecular Devices), and readings were normalized to the measurement obtained at day 0.

### Transwell migration and invasion assays

Twenty-four hours post-transfection, cells cultured at 80% confluence were treated with 10 µg/mL of mitomycin C (Sigma) to prevent proliferation prior performing the cell migration assay. Tumour cells were then resuspended in serum-free DMEM medium and seeded at a concentration of 10^4^ cells/200 µL/well in the upper chamber of a 24-well plate (Costar), while complete DMEM medium was used as a chemoattractant in the lower chamber of the well. After 24 h, cells in the upper chamber were removed with a cotton swab, and cells that had migrated through the porous membrane (8-µm diameter pore-size) were fixed in 100% ethanol, H&E stained, imaged under a Leica RMRB automatic upright microscope, and analysed with ImageJ 1.53k, Java 1.8.9_172 (64-bit) software. Tumour cell invasion experiments were conducted in 24-well plates in the same way as cell migration assays except 8-µm diameter pore-size polyethylene terephthalate (PET) membrane inserts were coated with Matrigel^®^ (BioCoat, Costar).

### Conditioned medium (CM) preparation from BC cells and mir-24-2-5p quantification

Six hours post-transfection, culture media of MIMIC-transfected NW1 (NW1/MIMIC-miR-24-2-5p, NW1/MIMIC-NegCtrl) and MCF7 (MCF7/MIMIC-miR-24-2-5p, MCF7/MIMIC-NegCtrl) BC cells were replaced with serum-free DMEM medium. BC cell-conditioned media (CM) were then collected after 24 h, centrifuged to remove cell debris, aliquoted, and stored at -80 °C until use for the osteoclastogenesis assay. Total RNA was extracted from 200uL of CM by using a NucleoSpin^®^ miRNA Plasm (Macherey-Nagel kit, Germany), accordingly to manufacturer instructions. MiR-24-2-5p expression levels were quantified as described above.

### Osteoclastogenesis assay

Osteoclastogenesis assay with human and murine monocytes was performed as previously described [10].

Briefly, primary human osteoclasts were differentiated from human peripheral blood mononuclear cells of healthy donors. The procedure was approved by the Ethical Committee of Campus Bio-Medico University of Rome (Prot. 21/15 OSS), and in accordance with the Declaration of Helsinki principles. Briefly, 1*10^4^ CD14 + monocytes resuspended in complete RPMI medium supplemented with M-CSF (25 ng/mL) and RANKL (50 ng/mL) were seeded in 96-well plates. After 72 h, cell culture medium was replaced with complete medium supplemented with M-CSF and RANKL, with or without BC cell-CM (1:16 volume dilution). Medium was replaced every 3 days. At day 12, cells were fixed and stained for TRAP activity (Sigma), or cell pellet was collected for gene expression analysis. Mature multinucleated osteoclasts (> 3 nuclei) were enumerated, and the total area covered by osteoclasts measured using a Nikon NIS-Elements microscope imaging software.

For murine osteoclastogenesis, bone marrow cells from tibiae and femora of 6/8-week-old OF1 male mice (Charles River) were flushed, centrifuged, resuspended in Ficoll^®^ Paque Plus (Cytiva), and further centrifuged to allow the isolation and enrichment of mononuclear cells. 1*10⁵ cells from the isolated mononuclear cell fraction were then seeded in 12-well plate wells, and cultured 24 h in α-MEM medium containing 10% (v/v) FCS (Invitrogen) with M-CSF (20 ng/mL). The next day, culture medium was replaced with a differentiation MEM-α medium containing 10% (v/v) FCS, M-CSF (20 ng/mL), and RANKL (10 ng/mL). For experiments conducted with BC cell-CM, mononuclear cells were continuously exposed to the CM (1:16 volume dilution), and mature multinucleated osteoclasts were then fixed after 7 days, stained for TRAP activity, and counted, or cell pellet was collected for gene expression analysis. For osteoclastogenesis experiments designed to measure endogenous miR-24-2-5p levels during the differentiation into osteoclasts of bone marrow-derived mononuclear cells treated with M-CSF and RANKL, bone marrow cells were fixed, TRAP stained, and counted at early and late time points, which correspond to immature and mature osteoclasts, respectively. Alternatively, cell pellets were collected at these early and late stages of osteoclast differentiation for gene expression analysis.

### TargetScan target prediction and ClueGO-based analysis

TargetScanHuman 7.0 software (https://www.targetscan.org/vert_70/, Accessed April 2021) was used to predict miR-24-2-5p targets in human transcriptome by searching for the presence of 8mer, 7mer, and 6mer sites that matched with miR-24-2-5p seed region. Top 250 predicted targets (arbitrary threshold) were used to perform a ClueGO-based analysis [18] using the ‘GO Biological Process’ database.

### RNA-seq of MIMIC-transfected BC cells

Thirty-six hours post-transfection, cell pellets from MIMIC-transfected NW1 (NW1/MIMIC-miR-24-2-5p, NW1/MIMIC-NegCtrl) or MCF7 (MCF7/MIMIC-miR-24-2-5p, MCF7/MIMIC-NegCtrl) BC cells from three independent experiments were collected and stored at -80 °C until use.

Total RNA was extracted using the RNeasy Mini Kit (Qiagen), according to the manufacturer’s recommendations. A DNase digestion step was performed during the RNA extraction using RNase-free DNase I Kit (Qiagen). RNA quantity and purity were evaluated using a 2100 Bioanalyzer (Agilent) and a Qubit RNA IQ Assay (ThermoFisher). RNA extracts (675 ng) were then used for RNA-seq library preparation with Poly-A enrichment.

A single-end RNA-seq was undertaken on the Illumina HiSeq™ 2500 platform in rapid run mode using the Illumina HiSeq™ Rapid Cluster Kit (Illumina, Inc., San Diego, CA, USA). Each sample had 15 million reads. RNA-seq data was aligned to GRCh38 human genome assembly using STAR v2.7.5c, and transcript quantification was performed using RSEM v1.3.1 as previously described [10]. Poorly expressed transcripts (< 0.5 counts per million in all samples) were eliminated for further analysis. Counts were normalized by weighted trimmed mean of M-values using TMM function of EdgeR Bioconductor package as previously described [10].

### Differential gene expression (DE) analysis and gene set enrichment analysis (GSEA)

DE analysis, based on negative binomial generalized linear models, was performed using EdgeR Bioconductor package to compare MIMIC-miRNA-24-2-5p transfected cells to MIMIC-negCTRL transfected cells [10].

GSEA analysis was performed using ClusterProfiler Bioconductor package on genes ranked by the fold change estimated in DE analysis. A manual annotation of gene networks has been performed based on the description of each gene network retrieved from GSEA website (https://www.gsea-msigdb.org/gsea/index.jsp, Accessed May 2022).

### Animal studies

Animal experiments conducted at the University of Sheffield (Sheffield, UK) were performed using 6-to-7-week-old female BALB/c fox/- *nude* mice (Charles River, Kent, UK) kept on a 12-h/12-h light/dark cycle with free access to food and water. NW1 cells transfected with MIMIC-miR-24-2-5p or negative control cells were injected into the left cardiac ventricle (5 × 10^4^ cells) of mice 72 h post-transfection. Tumour growth was monitored in anesthetized mice using an IVIS Lumina II system (Caliper Life Sciences, UK), following subcutaneous injection of D-Luciferin (Invitrogen, UK) to animals 4 minutes before imaging. Total blood was collected by cardiac puncture on the sacrifice day (9 days after intracardiac injection of tumour cells), aliquoted, and stored at -80 °C for downstream analysis by ELISA. An additional ex vivo imaging of hindlimbs and major organs by IVIS was performed at the autopsy to confirm site of metastases.

### Enzyme-linked immunosorbent assays (ELISA)

Serum concentrations of CTX-I (C-terminal telopeptide of type I collagen) and P1NP (pro-collagen type I N propeptide) were measured by ELISA using commercially available kits (E-EL-M3023 and E-EL-M3033, Elabscience), following manufacturers’ instructions.

### Statistical analysis

For combined ROC curve analysis (miR-24-5-5p with tumour grade), statistical significance was set at *p* < 0.05 (two-sided test), and a binary logistic regression was calculated using IBM SPSS Statistics. Broad institute software Morpheus was used to generate the plot. Survival analysis was performed by dividing patients into two groups [high (value > 0.1) and low (value <-0.1)] based on miR-24-2-5p expression (Z-score of 2^−ΔCT^). The Kaplan–Meier estimator was used to determine the relationship between miRNA expression and patient distant recurrence-free survival (only in bone). Differences in survival across the strata were calculated using a log-rank p-test.

All statistical analyses on experimental data were performed using Prism GraphPad 9.2.0 (GraphPad Software Inc., San Diego, CA, USA). Statistical significance was measured using parametric testing (Student’s t-test), assuming equal variance, and defined as p value (p) ≤ 0.05. All graphs represent mean ± standard error mean (SEM), *p ≤ 0.05, **p < 0.01, ***p < 0.001, ****p < 0.0001.

All in vitro experiments consisted of at least 3 independent biological repeats with appropriate controls.

For in vivo experiments, power calculations for animal experiments are based on our previous work [10, 19, 20]. In experiments conducted with NW1 cells, 70–80% of animals have metastasis. Assuming a power of 80% and a level of significance of 5%, we estimated that we will be able to measure a difference of 60% or greater with 9 animals per group, using a Mann-Whitney test.

For Cancer cell line Encyclopaedia (CCLE) analysis, information of miRNA-24-2-5p expression levels in 44 BC cell lines was retrieved from the public database. The violin plot was used to visualise the distribution of miRNA expression levels, which were reported as log2 read counts. Statistical significance was evaluated using the unpaired t-test, where p-values 0 < 0.05 were considered as significant.

For the TCGA, selected matched (RNA-seq and small RNA-seq) data sets from 736 patients were analysed. Pearson correlation was conducted to identify the association between miR-24-2-5p (RPM values, Z-score across the 736 samples) expression and target mRNA expression (TPM values, Z-score across the 736 samples).

## Results

### MiR-24-2-5p expression levels do not correlate with a specific BC subtype, and miR-24-2-5p is embedded in small extracellular vesicles released from BC cells

**Fig. 1:**
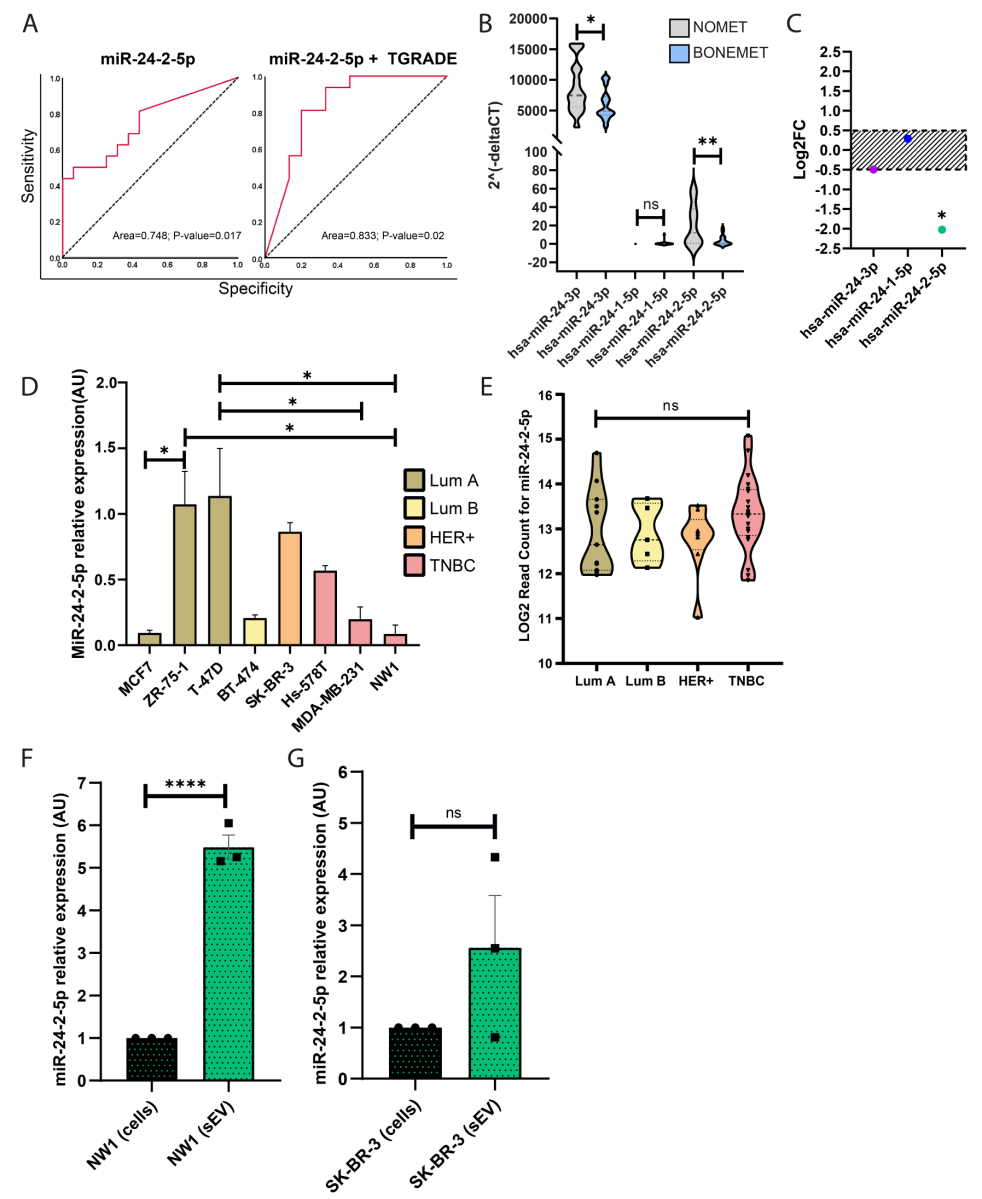
MiR-24-2-5p expression levels in the serum from BC patients, human BC cell lines, and small extracellular vesicles derived from BC cell lines. **A** Receiver operating characteristic (ROC) curves analysis for miR-24-2-5p to predict bone relapse, either alone (left panel) or in combination with patients’ tumour grade (TGRADE) (right panel). **B** Violin plot showing miR-24-3p, miR-24-1-5p and miR-24-2-5p expression levels in the serum from early-stage BC patients who did not relapse (NOMET, *n* = 16) *versus* BC patients who relapsed in bone (BONEMET, *n* = 16). **C** Comparison of the expression levels of miR-24-3p, miR-24-1-5p and miR-24-2-5p between NOMET and BONEMET groups. Only miR-24-2-5p expression levels showed a significant fold change (Log_2_FC≤-0.5, *p* = 0.02) between both groups. **D** Expression levels of miR-24-2-5p in different subtypes of human BC cell lines (luminal A, luminal B, HER+, TNBC). **E** Expression levels of miR-24-2-5p in the CCLE BC cell collection database (45 tumour cell lines sub-grouped into 4 BC subtypes). **F** Expression levels of miR-24-2-5p in small extracellular vesicles (sEVs) secreted from NW1 cells compared to the cells of origin. **G** Expression levels of miR-24-2-5p in sEVs secreted from SK-BR-3 cells compared to the cells of origin. Data are the mean ± SEM of three independent experiments, * *p* ≤ 0.05, ** *p* < 0.01, *** *p* < 0.001, **** *p* < 0.0001

We previously reported that miR-24-2-5p is downregulated in the serum from early-stage oestrogen receptor (ER)-positive BC patients who are at high risk of developing future (bone) metastasis (supplementary Fig. S1) [10]. We also showed that miR-24-2-5p has a good sensitivity and specificity (ROC curves analysis) to predict metastatic recurrence [10]. Interestingly, by combining serum miR-24-2-5p expression levels with patient’s tumour grade, we obtained a higher predictability for the risk of bone relapse (AUC = 0.833, *p* = 0.02) compared to miR-24-2-5p alone (AUC = 0.748, *p* = 0.017) (Fig. 1A). Kaplan–Meier survival analysis of the patients’ cohort (*N* = 48) revealed that the risk of bone metastasis had a trend to increase for patients with low miR-24-2-5p circulating levels (*P* = 0.053), compared to those who had high circulating levels (supplementary Fig. S2). We then evaluated circulating levels of miR-24-3p (the common form of miR-24 transcribed from both chromosomes 9 and 19), miR-24-1-5p (transcribed from chromosome 9 only), and miR-24-2-5p (transcribed from chromosome 19 only) using the serum from early-stage BC patients who did not relapse (NOMET, *n* = 16) compared to those who first relapsed in bone (BONEMET, *n* = 16) (Fig. 1B). MiR-24-3p was more expressed in the serum than miR-24-1-5p and miR-24-2-5p forms (Fig. 1B), the latter having very low detectable levels (supplementary Fig. S3). Both miR-24-3p and miR-24-2-5p showed a significative reduction of their expression levels in the BONEMET group compared to NOMET (Fig. 1B); however, only miR-24-2-5p showed a statistically significant decreased fold change (Log_2_FC≤ -0.5) between the two groups. (Fig. 1C). Overall, these results indicated that low circulating levels of miR-24-2-5p at baseline were associated with a higher risk to develop future (bone) metastasis in early-stage ER-positive BC patients.

We then examined if miR-24-2-5p expression varied across BC subtypes. MiR-24-2-5p expression levels varied greatly from one BC cell line to another, with the highest expression levels being observed in T-47D cells (luminal A), and the lowest in NW1 (TNBC, triple negative BC) and MCF7 (luminal A) cells (Fig. 1D). This observation extended to the analysis of 45 additional human BC cell lines of the ‘Cancer Cell Line Encyclopaedia’ (CCLE) BC cell collection database (Fig. 1E), indicating therefore that miR-24-2-5p expression levels did not correlate with any specific BC subtype and/or tumour cell aggressiveness trait.

Finally, since miR-24-2-5p was detected in the serum of BC patients, we questioned if miR-24-2-5p could be embedded in tumour cell-derived small extracellular vesicles (sEVs), which are known to have an important role in miRNA stability in the circulation as well as a functional role in cell-to-cell communication [21]. We therefore isolated sEVs from the tumour cell conditioned medium (CM) of NW1 and SK-BR-3 BC cell lines (having low and high endogenous miR-24-2-5p levels, respectively). MiR-24-2-5p was detected in sEVs secreted from NW1 (Fig. 1F) and SK-BR-3 cells (Fig. 1G). Moreover, there was a 5-fold enrichment of miR-24-2-5p in sEVs from NW1 cells compared to the cells from which they were derived (Fig. 1F).

### Forced miR-24-2-5p expression in BC cells reduced proliferation, migration, and invasion in vitro

**Fig. 2:**
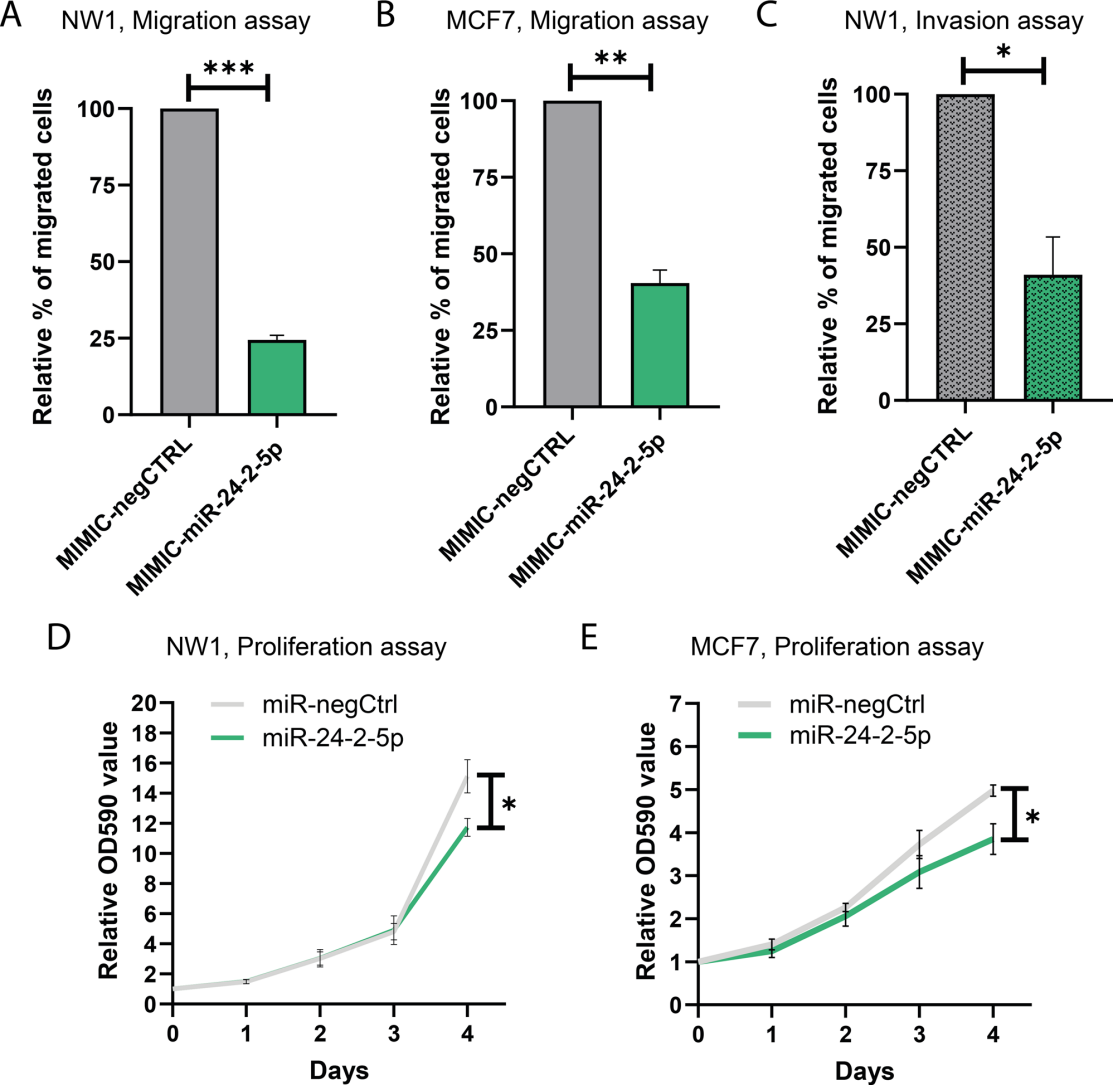
Effects of miR-24-2-5p overexpression on migration, invasion, and proliferation of BC cells. **A** Migration assay at 24 h using transiently transfected NW1 cells overexpressing miR-24-5p (MIMIC-miR-24-2-5p) or the negative control mimic (MIMIC-negCTRL). **B** Same as in (A) for the migration of transiently transfected MCF7 cells (MIMIC-negCTRL, MIMIC-miR-24-2-5p). **C** Invasion assay at 24 h using transiently transfected NW1 cells (MIMIC-negCTRL, MIMIC-miR-24-2-5p). **D** Proliferation assay using transduced NW1 cells stably overexpressing miR-24-5p (MIMIC-miR-24-2-5p) or the negative control mimic (MIMIC-negCTRL). **E** Same as in (D) for the proliferation of transduced MCF7 cells (miR-negCTRL, miR-24-2-5p). Data are the mean ± SEM of 3 independent experiments. * *p* ≤ 0.05, ** *p* < 0.01, *** *p* < 0.001

Since miR-24-2-5p expression levels in MCF7 and NW1 (a *luc2*-positive MDA-MB-231 subpopulation) cells were lower than in other BC cell lines, these cells appeared as useful cellular models for gain-of-function experiments designed to better understand a possible implication of miR-24-2-5p in the progression of BC metastasis. Forced overexpression of miR-24-2-5p in NW1 and MCF7 cells was obtained following transient transfection with miRNA mimics for miR-24-2-5p (MIMIC-miR-24-2-5p) or negative control miRNA mimics (MIMIC-negCTRL) (supplementary Figs. S4A and C). NW1 and MCF7 cells were also transduced to stably express miR-24-2-5p (NW1/miR-24-2-5p, MCF7/miR-24-2-5p) compared to control cells (NW1/miR-negCTRL, MCF7/miR-negCTRL) (supplementary Fig. S4B, and Fig. 4D). We then used these cells to conduct functional cell-based assays (migration, invasion, proliferation). In a short-term experiment (24 h), the acute overexpression of miR-24-2-5p by using miRNA mimics considerably decreased migratory properties of both NW1 and MCF7 transfected cells compared to their respective negative controls (*p* < 0.001 and *p* < 0.01, respectively) (Fig. 2A and B). The effect of miR-24-2-5p overexpression on invasion was tested using NW1 cells, which are known to be highly invasive [22]. MiR-24-2-5p overexpression decreased NW1 cell invasive properties compared to control (*p* < 0.05) (Fig. 2C). Next, we used stably overexpressed miR-24-2-5p (NW1/miR-24-2-5p, MCF7/miR-24-2-5p) or control (NW1/miR-negCTRL, MCF7/miR-negCTRL) cells to assess cell proliferation in a relatively longer-term experiment (4 days). In both cellular models, overexpression of miR-24-2-5p decreased cell proliferation in comparison to control cells (NW1, *p* < 0.01; MCF7, *p* < 0.05) (Fig. 2D and E).

We also tested the effect of miR-24-2-5p inhibition in human BC cell lines expressing high endogenous miR-24-2-5p levels. Downregulation of miR-24-2-5p was obtained following transient transfection of a miRNA inhibitor for miR-24-2-5p (INHIB-miR-24-2-5p) in ZR-75-1, T-47D, and SK-BR-3 cell lines. As assessed by real-time qPCR, miR-24-2-5p expression levels were drastically decreased in these BC cells transfected with INHIB-miR-24-2-5p compared to their relative controls transfected with a negative control inhibitor (INHIB-negCTRL) (supplementary Fig. S5). This substantial reduction of miR-24-2-5p expression did not, however, lead into a stimulatory effect of the proliferation (supplementary Fig. S6) and migration (supplementary Fig. S7) of ZR-75-1, T-47D, and SK-BR-3 cell lines. These data indicated that the downregulation of miR-24-2-5p expression levels alone was not sufficient to modulate the intrinsic proliferative and migratory properties of these BC cell lines. This agreed with the observation that miR-24-2-5p did not correlate with BC cell aggressiveness (Fig. 1E). Conversely, in BC cells expressing low endogenous miR-24-2-5p levels and having high intrinsic proliferative, migratory and invasive properties, the restoration of miR-24-2-5p expression levels led to a significant inhibitory effect on cell proliferation, migration and invasion in vitro (Fig. 2).

### Transcriptomic analyses of BC cells revealed a set of genes specifically modulated by miR-24-2-5p

**Fig. 3:**
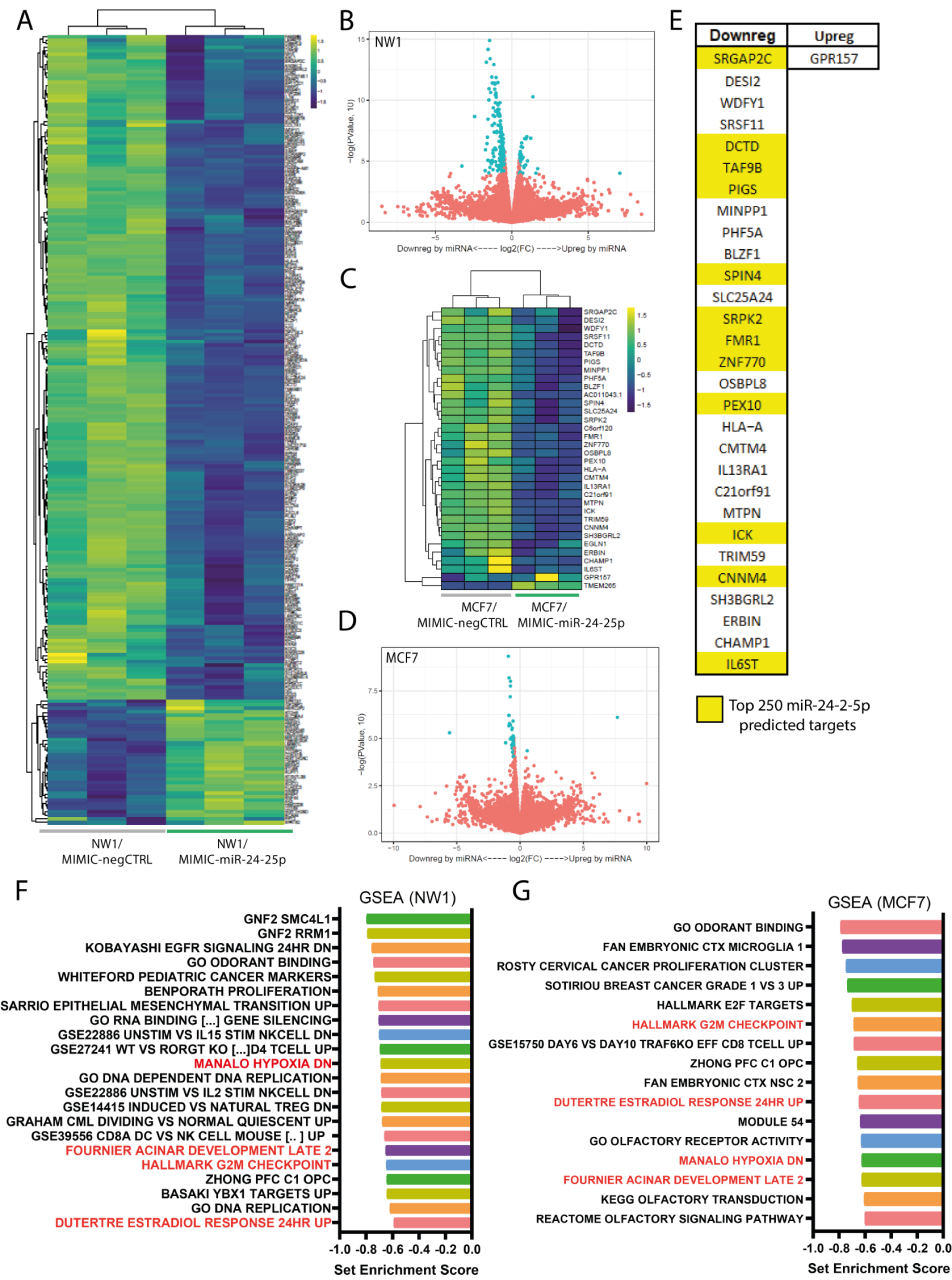
RNA-sequencing analyses on miR-24-2-5p-overexpressing BC cells. **A** Heatmap for transcripts modulated by miR-24-2-5p in NW1 cells. **B** Volcano plot with differentially expressed transcripts modulated by miR-24-2-5p in NW1 cells. **C** Heatmap for transcripts modulated by miR-24-2-5p in MCF7 cells. **D** Volcano plot with differentially expressed transcripts modulated by miR-24-2-5p in MCF7 cells. **E** Common transcripts modulated by miR-24-2-5p in both NW1 and MCF7 cells. In yellow, transcripts that were predicted direct targets of miR-24-2-5p. **F** GSEA on modulated transcripts in RNA-seq data from NW1 cells transiently transfected with MIMIC-miR-24-2-5p *versus* MIMIC-negCTRL. **G** GSEA on modulated transcripts in RNA-seq data from MCF7 cells transiently transfected with MIMIC-miR-24-2-5p *versus* MIMIC-negCTRL. Common gene sets between NW1 and MCF7, GSEA analyses are highlighted in red

To evaluate whole transcriptomic changes due to the overexpression of miR-24-2-5p in BC cells at an early time-point, we performed RNA-sequencing (RNA-seq) on both NW1 and MCF7 cells at 36 hours post-mimic transfection. A total of 222 transcripts were modulated by miR-24-2-5p overexpression in NW1 cells compared to controls (Fig. 3A and B), with most of them (187 transcripts) being downregulated. A similar trend was observed in MCF7 cells, where a lower number (34 transcripts) were regulated by miR-24-2-5p overexpression compared to controls, almost all of them (32 transcripts) being downregulated (Fig. 3C and D). In order to focus on miR-24-2-5p-mediated molecular pathways shared between NW1 and MCF7 cells, we overlapped the results obtained from the two cell lines to identify common elements. A total of 30 transcripts (29 downregulated, 1 upregulated) were shared between the two screens (Fig. 3E). Moreover, 12 (out of 29) downregulated transcripts were among the top 250 predicted targets of miR-24-2-5p, using TargetScan software (Fig. 3E, and supplementary Table S2). Gene set enrichment analysis (GSEA) was performed on RNA-seq data from NW1 and MCF7 screens. For the transcripts modulated by miR-24-2-5p in NW1 cells, a total of 22 gene pathways were significantly enriched (Set Enrichment Score < 0.5) (Fig. 3F, and supplementary Table S3). Among the top 10 more (negatively) enriched sets, we identified ‘*Benporath proliferation*’ and ‘*Sarrio Epithelial Mesenchymal Transition UP*’ gene sets that are both involved in human BC cell proliferation, and ‘*Hallmark G2M checkpoint*’ set that includes genes involved in the progression through the cell division cycle, the latter being indirectly related to cell proliferation. For the transcripts modulated by miR-24-2-5p in MCF7 cells, a total of 16 gene pathways were significantly enriched (Set Enrichment Score < 0.5) (Fig. 3G, and supplementary Table S3). Of note, this analysis pointed out the ‘*Module 54’*, which is a cancer-related set of genes mainly upregulated in BC, and being negatively enriched, which suggested a possible role of miR-24-2-5p in BC progression. Moreover, we identified 4 shared gene sets between NW1 and MCF7 GSEA analyses (Fig. 3F and G), that were either downregulated during hypoxia, expressed by normal mammalian cells during acinar development, upregulated in MCF7 cells after estradiol treatment, and/or involved in cell cycle.

**Fig. 4:**
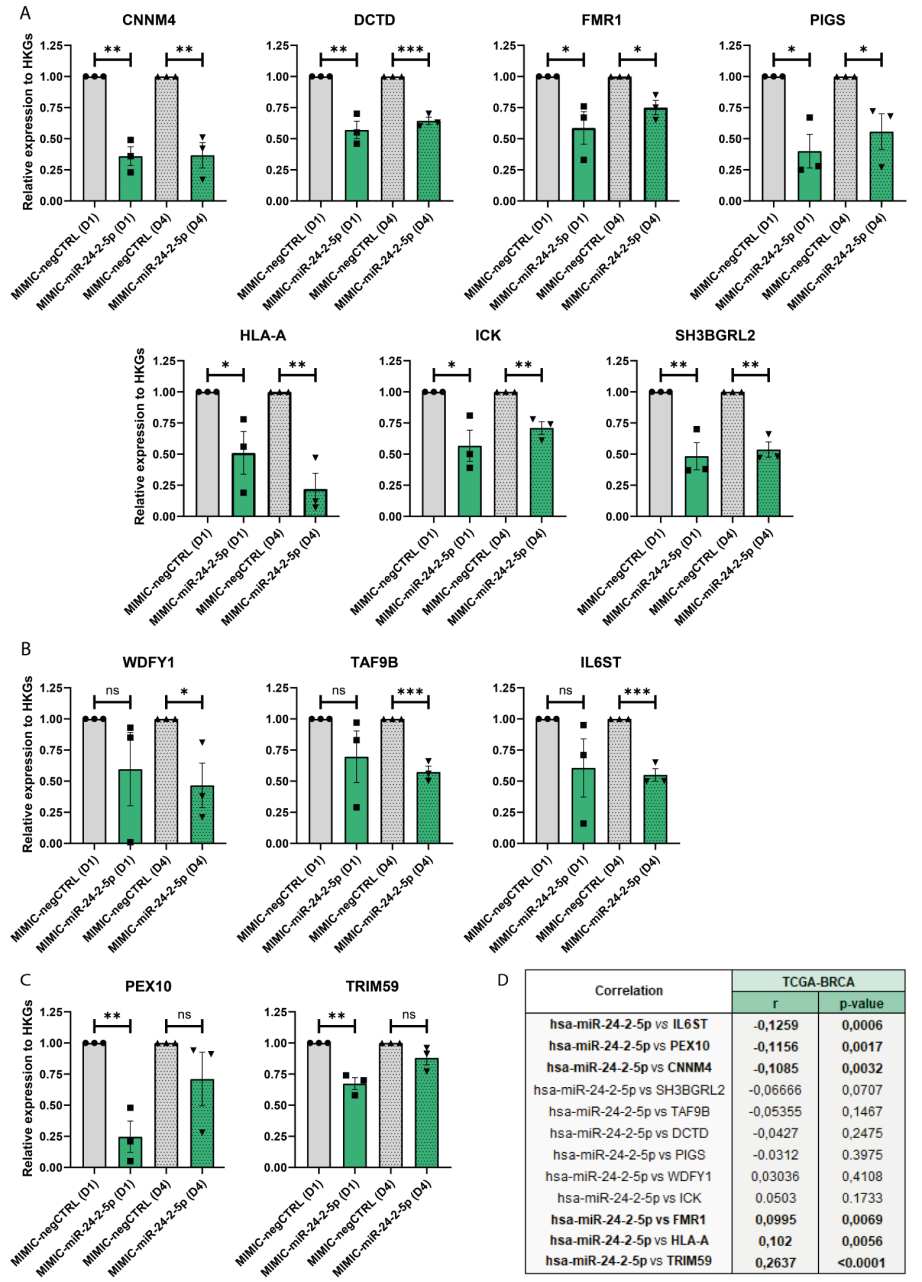
Real-time qPCR validation of transcripts downregulated by miR-24-2-5p overexpression. **A** Validated transcripts downregulated upon miR-24-2-5p overexpression at both early (day 1, D1) and late (day 4, D4) in NW1 cells transiently transfected with MIMIC-miR-24-2-5p *versus* MIMIC-negCTRL. **B** Validated transcripts downregulated only at D4 in NW1 cells transiently transfected with MIMIC-miR-24-2-5p *versus* MIMIC-negCTRL. **C** Validated transcripts downregulated only at D1 in NW1 cells transiently transfected with MIMIC-miR-24-2-5p *versus* MIMIC-negCTRL. **D** Correlations of miR-24-2-5p expression and the expression of each validated transcript in 736 primary BC tissues from the TGCA-BRCA database. Significant correlations are highlighted. Data are the mean ± SEM of 3 independent experiments. * *p* ≤ 0.05, ** *p* < 0.01, *** *p* < 0.001, **** *p* < 0.0001

Based on literature [23], our RNA-seq and GSEA data, and our target prediction with TargetScan software, we decided to validate by real-time qPCR some of the transcripts that were downregulated following miR-24-2-5p overexpression in both NW1 and MCF7 cells. Thus, NW1 cells were transiently transfected with a miR-24-2-5p mimic (MIMIC-miR-24-2-5p) or a negative control mimic (MIMIC-negCTRL), and then collected at two different time points (day 1 and 4) to evaluate early and late gene expression levels upon miR-24-2-5p overexpression. CNNM4, DCTD, FMR1, PIGS, HLA-A, ICK and SH3BGRL2 transcripts were significantly downregulated by miR-24-2-5p overexpression in comparison to control at both 1- and 4-days post-transfection (Fig. 4A); WDFY, TRAF9B and IL6ST transcripts were downregulated on day 4 only (Fig. 4B); PEX10 and TRIM59 transcripts were downregulated on day 1 only (Fig. 4C). No differences in gene expression was observed for MINPP1 and ZNF770 transcripts (supplementary Fig. S8). Overall, most of the transcripts downregulated upon miR-24-2-5p overexpression in NW1 and MCF7 cells in our RNA-seq data were validated by real-time qPCR at an early and/or late time-point.

To evaluate if there was a correlation between miR-24-2-5p and the modulated transcripts (CNNM4, DCTD, FMR1, PIGS, HLA-A, ICK, SH3BGRL2, WDFY, TRAF9B, IL6ST, PEX10, TRIM59) in BC tissues from patients, we examined their expression levels in primary tumours (*n* = 736) by interrogating the TCGA-BRCA dataset. We found that IL6ST, PEX10, and CNNM4 transcripts had a significant but poor inverse correlation in relation to miR-24-2-5p expression levels (Fig. 4D). A trend of inverse correlation with miR-24-2-5p was noticed for SH3BGRL2, while no changes or a poor positive correlation were observed for the other transcripts (Fig. 4D). These results only suggested that miR-24-2-5p might be involved in the downregulation of IL6ST, PEX10, and CNNM4 at the breast tumour tissue level. However, it needs to be considered that all transcripts (showing an inverse or a positive correlation) are likely to be regulated through multiple pathways, indicating that our results remained merely indicative of the role of miR-24-2-5p in more complex primary BC tissues.

### Forced miR-24-2-5p expression in metastatic BC cells reduces bone metastasis in vivo

**Fig. 5:**
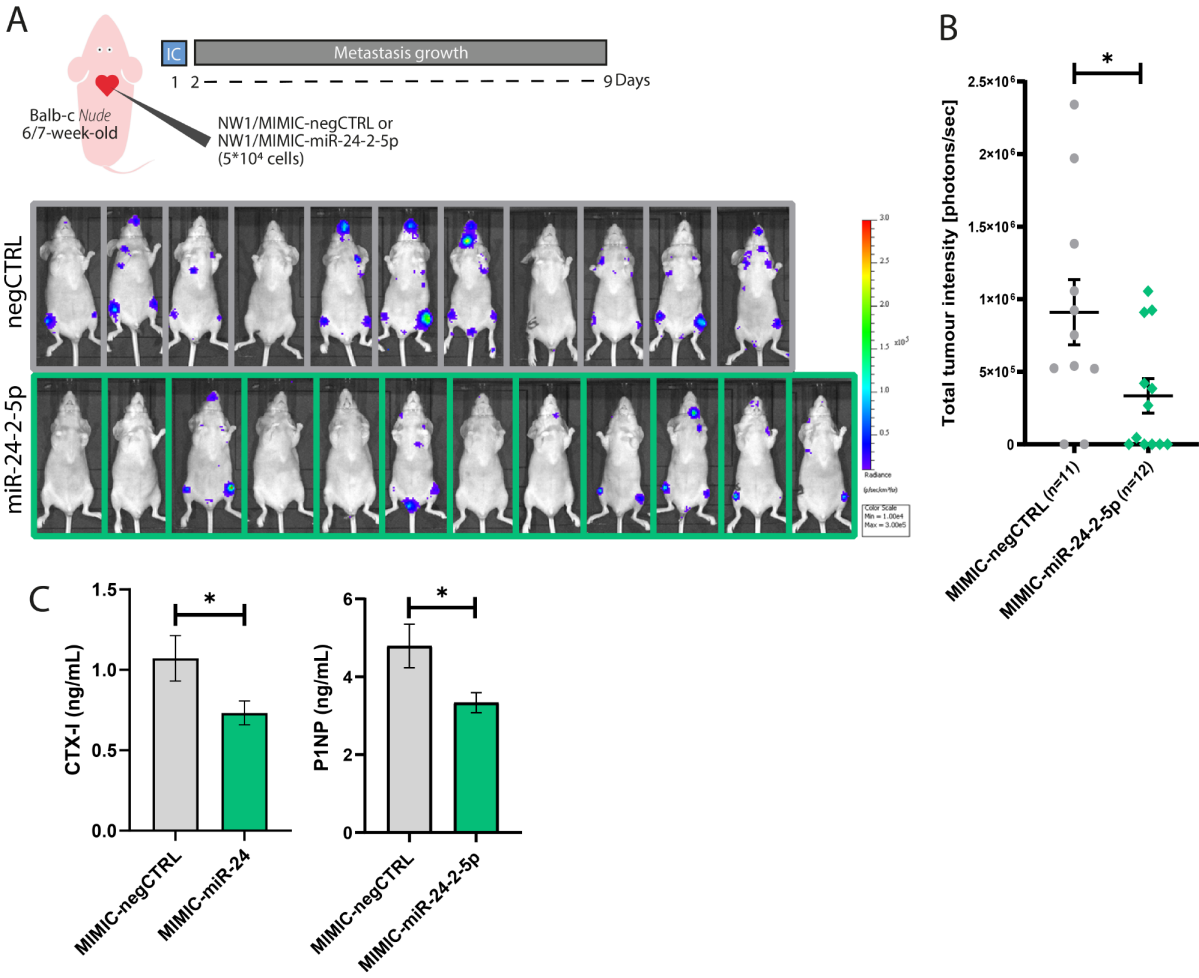
Experimental model of bone metastasis to evaluate the effect of miR-24-2-5p on early BC dissemination in vivo. **A** Schematic representation of the protocol aimed to evaluate the impact of miR-24-2-5p overexpression on the early stages of (bone) metastasis formation in animals. The control group of mice that received an intracardiac injection of control NW1 cells (upper panel, gray) showed metastasis formation, mainly in bones (e.g., jaws, hind limbs, and shoulders), 7 days post tumour cell injection, as judged by BLI. Instead, the experimental group that received an intracardiac injection of miR-24-2-5p-overexpressing NW1 cells (lower panel, green) showed a drastic reduction or absence of metastases. **B** Total bioluminescence intensity of each mouse that belonged to the control (MIMIC-negCTRL) or experimental (MIMIC-miR-24-2-5p) group was quantified as a readout of tumour burden in animals at day 7 post injection. **C** Circulating CTX-I (bone resorption marker) and P1NP (bone formation marker) levels in control (MIMIC-negCTRL) and experimental (MIMIC-miR-24-2-5p) group at day 9 (sacrifice day). Data are shown as mean value ± SEM. * *p* ≤ 0.05

We previously reported that the intracardiac injection of NW1 cells in mice is a reliable model to study early and late stages of bone metastasis formation [10, 19]. Transient transfection of NW1 cells with miR-24-2-5p mimics was chosen because *(1)* a higher miR-24-2-5p overexpression was obtained compared to miR-24-2-5p levels expressed in transduced NW1/miR-24-2-5p cells (supplementary Fig. S4), and *(2)* transfected cells still retain miR-24-2-5p mimics when circulating in the blood stream until they seed and start to proliferate in bone [10]. Thus, NW1 cells were transiently transfected with a miR-24-2-5p mimic (MIMIC-miR-24-2-5p) or a negative control mimic (MIMIC-negCTRL) prior to their intracardiac injection in immunocompromised mice, and then the tumour formation in mice was monitored over time using in vivo bioluminescence imaging (BLI) (Fig. 5A). As judged by BLI, NW1 cells overexpressing miR-24-2-5p had a reduced ability to form metastasis in vivo (*p* < 0.05), especially in hind limbs and jaws (Fig. 5A and B).

Once in bone, BC cancer cells disrupt normal bone homeostasis, altering bone formation and increasing bone resorption, which often results in bone destruction [2]. In animal models of BC bone metastasis, the presence of osteolytic lesions is usually detected by microcomputed tomography or radiography [2]. However, since our animal study was designed to evaluate the onset of bone metastasis formation (i.e., 9 days after tumour cell inoculation in animals), at which time there is no radiographic evidence of osteolytic lesions, instead, we measured circulating levels of CTX-I, a marker for bone resorption, and P1NP, a marker for bone formation. In the clinic, high baseline P1NP and CTX-I serum levels in early-stage BC patients are predictive of future bone recurrence, indicating that there is a relationship between accelerated baseline bone turnover and subsequent distant recurrence events [24]. We found that, compared to animals injected with control NW1 cells, mice injected with miR-24-2-5p-overexpressing NW1 cells had reduced circulating CTX-I levels (*p* = 0.0386) (Fig. 5C), and these low CTX-I levels were comparable to those observed in naïve mice of the same age (supplementary Fig. S9). Furthermore, animals injected with miR-24-2-5p-overexpressing cells had low P1NP circulating levels (*p* = 0.0168), compared to control animals (Fig. 5C), indicating that there was not only a reduction in bone resorption as indicated by lower CTX-I levels, but also a decrease in bone formation, which led to an overall decrease of bone turnover in these mice.

Altogether, these data suggested that miR-24-2-5p had a protective effect by reducing the metastatic potential of circulating BC cells in vivo.

### The conditioned medium from miR-24-2-5p-overexpressing BC cells reduces osteoclastogenesis in vitro

**Fig. 6:**
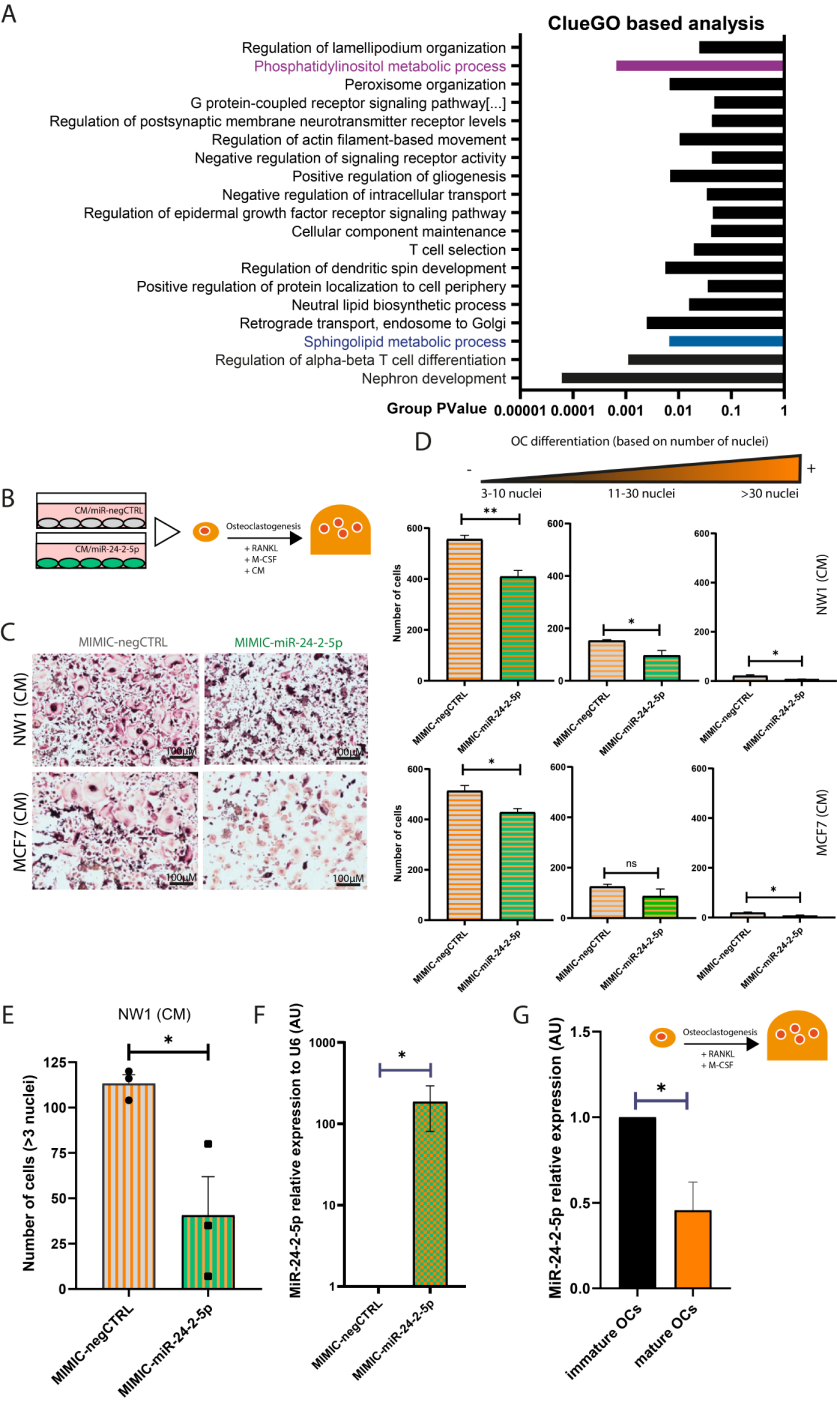
Effect of the conditioned medium (CM) from miR-24-2-5p-overexpressing BC cells on osteoclast differentiation. **A** ClueGO-based analysis conducted on the top 250 predicted targets (TargetScan) for miR-24-2-5p. 18 GO biological process networks (groups) with the relative p-value are presented. Two groups of high relevance for osteoclastogenesis are highlighted. **B** Schematic representation of the experimental protocol with human blood-derived monocytes cultured in the presence of differentiating factors (RANKL, M-CSF) and the conditioned medium (CM) from BC cells overexpressing or not overexpressing miR-24-2-5p. **C** Representative figures of differentiated, TRAP-positive osteoclasts after 12 days in culture in the presence of CM from control (MIMIC-negCTRL) or miR-24-2-5p-overexpressing (MIMIC-miR-2-5p) NW1 or MCF7 cells (upper and lower panels, respectively). **D** Histograms showing the number of TRAP-positive osteoclasts in the presence of CM from control (MIMIC-negCTRL) or miR-24-2-5p-overexpressing (MIMIC-miR-2-5p) NW1 (upper panel) and MCF7 (lower panel) cells. Mature osteoclasts were classified into 3 groups: between 3 and 10 nuclei, from 11 to 30 nuclei, and more than 30 nuclei. **E** Effect of CM from NW1 cells overexpressing miR-24-2-5p (MIMIC-miR-24-2-5p) or a negative control mimic (MIMIC-negCTRL) on the differentiation of murine bone marrow-derived osteoclasts. Plot showed the number of multinucleated (> 3 nuclei) TRAP-positive osteoclasts for each group. **F** MiR-24-2-5p expression levels in osteoclasts cultured with CM from NW1 cells transfected with miR-24-2-5p mimics (MIMIC-miR-24-2-5p) or negative control miRNA mimics (MIMIC-negCtrl). Cell pellets were collected at day 7 of the osteoclastogenesis protocol. Relative expression levels of miR-24-2-5p compared to the housekeeping gene U6 were calculated by the 2^−ΔΔCT^ method. **G** MiR-24-2-5p expression levels in immature and mature murine osteoclasts (the categorization was based on TRAP-positivity and gene expression analysis of markers related to osteoclastogenesis). Relative expression levels of miR-24-2-5p compared to the housekeeping gene U6 were calculated by the 2^−ΔΔCT^ method. Data are the mean ± SEM of 3 independent experiments. * *p* ≤ 0.05, ** *p* < 0.01

MiRNAs produced by metastatic BC cells can regulate gene expression of surrounding bone cells and their activities, thus playing an additional role during bone metastases formation [2, 25]. In this respect, BC cells secrete soluble factors that accelerate osteoclast differentiation, leading to the formation of osteolytic bone metastases [8]. Here, we used the top 250 predicted direct targets of miRNA-24-2-5p (supplementary Table S2) to conduct a ClueGO-based analysis, and found that miR-24-2-5p predicted targets were mainly involved in 4 distinct GO-biological processes: ‘nephron development’, ‘regulation of alpha-beta T cell differentiation’, ‘sphingolipid metabolic process’, and ‘phosphatidylinositol metabolic process’ (Fig. 6A, and supplementary Fig. S10 and Table S4). In particular, we noticed that a group of direct predicted targets of miR-24-2-5p (SOCS2, SOCS4, TEK, UVRAG, EFR3A, LPGAT1, MTMR6, PIGS, PIP4P1) that constituted the ‘sphingolipid metabolic process’ and ‘phosphatidylinositol metabolic process’ networks have previously been shown to be involved in osteoclastogenesis [26–29]. After having observed that miR-24-2-5p overexpression in NW1 cells reduced bone resorption in vivo (Fig. 5C), we therefore asked whether miR-24-2-5p had any influence on osteoclast differentiation.

We first tested the effect of conditioned media (CM) collected from NW1 or MCF7 cells transfected with MIMIC-miR-24-2-5p or a negative control mimic (MIMIC-negCTRL) on the osteoclast differentiation of human peripheral blood mononuclear cells exposed to osteoclast differentiating factors (M-CSF, RANKL) (Fig. 6B). The CM from miR-24-2-5p-overexpressing NW1 and MCF7 cells reduced osteoclast differentiation and activity (evaluated by TRAP staining) promoted by M-CSF and RANKL, compared to the CM from cells overexpressing a negative control miRNA mimic (Fig. 6C and D, and supplementary Fig. S11). Specifically, CM from miR-24-2-5p-overexpressing NW1 and MCF7 cells almost completely inhibited the formation of osteoclasts with more than 30 nuclei (Fig. 6D), the latter being those that are the most active to resorb bone [30]. This inhibitory effect of the CM from miR-24-2-5-overexpressing NW1 and MCF7 cells on human osteoclastogenesis was accompanied by a decreased gene expression of markers associated with osteoclast differentiation, including ACP5 (Tartrate-Resistant Acid Phosphatase 5, also known as TRAP), CTSK (Cathepsin K, an osteoclast-derived cysteine protease enabling degradation of the collagenous matrix), CALCR (calcitonin receptor), and MMP-9 (Matrix Metallopeptidase 9, an osteoclast-derived metalloproteinase mediating matrix degradation) (supplementary Fig. S12).

We next conducted similar osteoclastogenesis experiments using murine bone marrow-derived monocytes exposed to M-CSF and RANKL. As for human osteoclastogenesis, we found that the CM from miR-24-2-5p-overexpressing NW1 cells reduced murine osteoclast differentiation induced by M-CSF and RANKL compared to the CM from NW1 cells overexpressing a negative control miRNA mimic (Fig. 6E). Interestingly, miR-24-2-5p expression levels were drastically increased in the CM from miR-24-2-5p-overexpressing NW1 cells compared to the CM from NW1 control cells (supplementary Fig. S13), and also in osteoclasts cultured with the CM from miR-24-2-5p-overexpressing NW1 cells compared to osteoclasts cultured with the CM from NW1 control cells (Fig. 6F), indicating an uptake of tumour-derived miR-24-2-5p by murine osteoclasts likely responsible of the reduction in the osteoclast differentiation.

To further investigate the role of miR-24-2-5p during osteoclastogenesis, we also measured endogenous miR-24-2-5p expression levels during the differentiation into osteoclasts of murine bone marrow-derived monocytes treated with differentiating factors (M-CSF, RANKL). The osteoclast differentiation status (defined as immature or mature) was evaluated by TRAP staining (supplementary Fig. S14) and real-time qPCR gene expression analysis of markers associated with osteoclast differentiation and cytoskeleton morphology (supplementary Fig. S15). We found that endogenous miR-24-2-5p expression levels were significantly reduced in mature osteoclasts compared to their immature counterparts (Fig. 6G). These observations indicated that, under more physiological conditions, a downregulation of miR-24-2-5p expression levels was occurring during osteoclast differentiation in vitro.

Collectively, these results showed the importance of the inhibitory role of tumour-derived miR-24-2-5p during human and murine osteoclast differentiation in vitro.

## Discussion

**Fig. 7:**
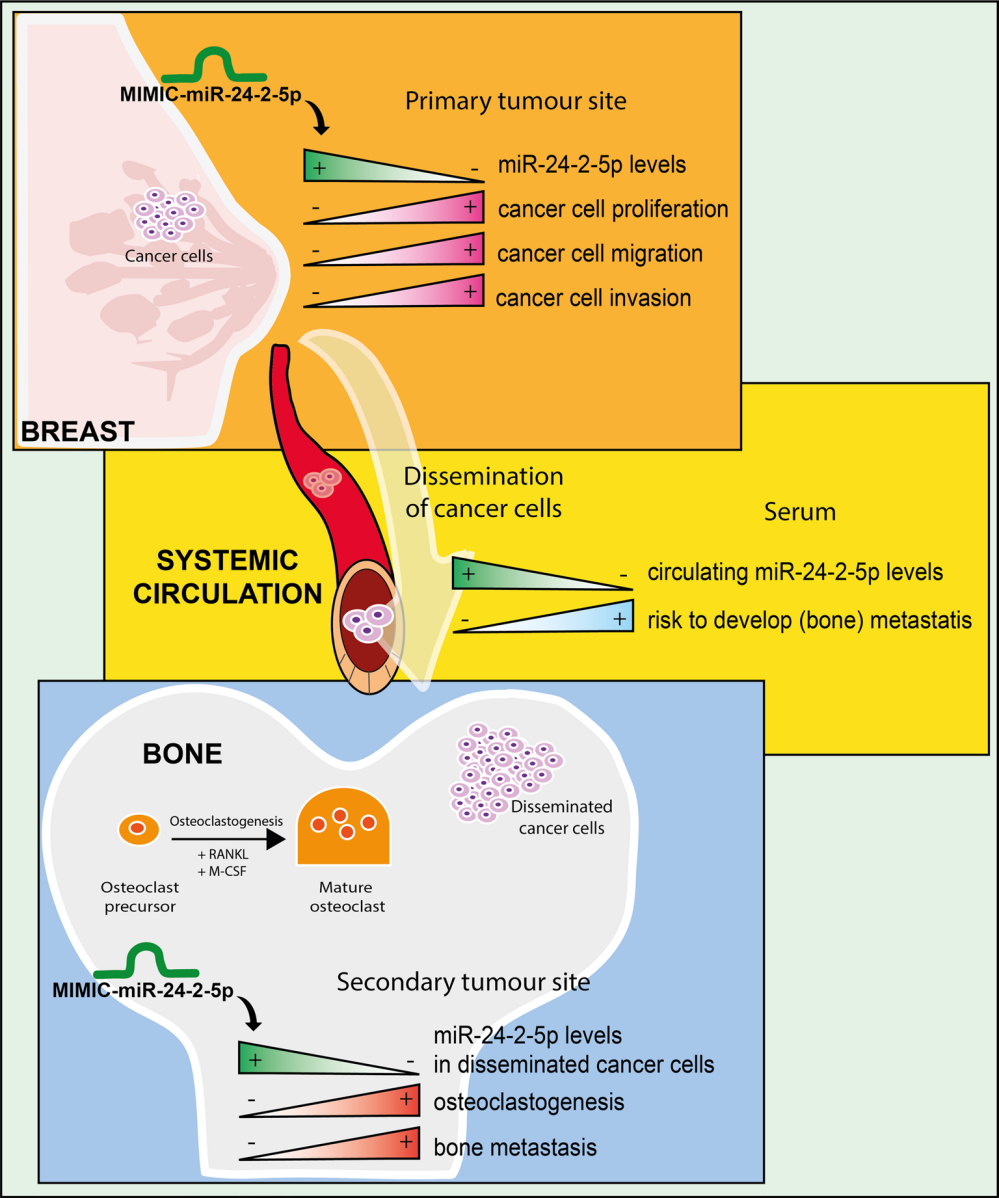
Schematic representation of the role of miR-24-2-5p during breast cancer progression and bone metastasis formation. In invasive human BC cells, forced miR-24-2-5p expression reduces cell malignant traits in vitro (upper panel). Low miR-24-2-5p expression levels in serum of early-stage BC patients are associated with a high risk to develop (bone) metastasis (middle panel). Forced expression of miR-24-2-5p in disseminated BC cells in bone has a protective effect in vivo by reducing the metastatic potential of circulating BC cells and decreasing bone turnover (lower panel). Overall, miR-24-2-5p has a protective role in the first events of BC bone metastasis formation, reducing malignant BC cell traits, tumour cell dissemination in bone, and the differentiation of monocytes into mature osteoclasts

Metastasis to the bone is common in advanced stage of BC [2], and miRNAs play important roles in regulating the progression of BC metastatic disease [2, 8]. We have previously shown that low circulating miR-24-2-5p levels in the serum at baseline (time of surgery) from early-stage BC patients are associated with a high risk to develop future bone metastasis [10]. Experimentally, miR-24-2-5p decreases growth of human BC xenografts in animals and tumour cell survival in vitro [12], suggesting that miR-24-2-5p might act as a tumour suppressor. However, a potential tumour suppressive role for miR-24-2-5p in BC bone metastasis has never been explored. Here, we demonstrated that miR-24-2-5p attenuates breast-to-bone metastasis. Specifically, we showed that miR-24-2-5p overexpression in BC cells reduces their proliferative and migratory/invasive properties in vitro, and bone metastasis formation in vivo. Additionally, we showed that miR-24-2-5p overexpression in metastatic BC cells also has an impact on bone homeostasis, inhibiting osteoclast differentiation in vitro and bone resorption in vivo. Thus, miR-24-2-5p employs multiple inhibitory mechanisms to reduce the formation of BC bone metastasis (Fig. 7).

In this study, we chose to overexpress miR-24-2-5p in human ER-positive (MCF7) or triple negative (NW1) BC cells that express low endogenous miR-24-2-5p levels because *(1)* circulating levels of this miRNA were extremely low in the serum of BC patients who develop bone metastases, and *(2)* miR-24-2-5p expression levels did not correlate with any specific BC subtype (Fig. 1). In order to reduce the risk of technical bias, miR-24-2-5p overexpression in tumour cells was obtained using two different overexpressing systems: (i) miRNA mimics to force a potent yet transient overexpression, and (ii) the stable overexpression. The use of miRNA mimics is an efficient and trustable technique to upregulate miRNAs, with a clinical potential [17, 25, 31, 32]. Moreover, the overexpression strategy allowed us to study the role of miR-24-2-5p using an animal model of bone metastasis in which transfected NW1 cells were injected intracardially to mice. In MCF-7 and NW1 cells expressing low endogenous miR-24-2-5p levels, increased miR-24-2-5p expression levels led to a significant inhibitory effect on cell proliferation, migration, and invasion in vitro (Fig. 2). A similar inhibitory effect of miR-24-2-5p on DU145 prostate cancer cell proliferation has previously been reported, when overexpressing a miRNA mimic [33]. Our results are also complementary to those observed in primary BC progression where miR-24-2-5p overexpression decreases growth of tumour xenografts [12], thus reinforcing the idea that miR-24-2-5p acts as a tumour suppressor. We also tested the effect of miR-24-2-5p inhibition in human BC cell lines (ZR-75-1, T-47D, SK-BR-3) with high miR-24-2-5p endogenous levels following transient transfection with a miRNA inhibitor for miR-24-2-5p. The downregulation of miR-24-2-5p did not modulate proliferative and migratory properties of these BC cell lines (supplementary Figs. S6 and S7). It is likely that the downregulation of miR-24-2-5p alone was not sufficient to modulate proliferative and migratory capacities of human BC cell lines that have not already acquired those properties by expressing epithelial-to-mesenchymal-related transcription factors. Comparable results were obtained with miR-24-3p, a tumour promoter, whose overexpression in human basal MDA-MB-435 and MDA-MB-468 BC cell lines only modestly stimulates tumour growth in vitro [23]. Furthermore, we previously reported that Twist1-induced miR-10b overexpression in human basal MDA-MB-231/B02 BC cells does not further modify their mesenchymal appearance nor their growth in vitro [34]. For these reasons, we did not perform additional experiments using the miR-24-2-5p-downregulation strategy, but we focussed on the effect of miR-24-2-5p expression restoration in BC cells instead. Altogether, our experimental results suggest that the restoration of protective levels of miR-24-2-5p in BC cells at the primary site could be a potential therapeutic approach to reduce metastasis by impeding BC cells to evade the primary site and then colonise distant organs, such as bone. In this respect, a specific delivery system of miR-24-2-5p mimics in artificial liposomes [35] or nanoparticles [36] might represent a promising therapeutic opportunity to target the very first events of BC progression.

To further study the role of miR-24-2-5p in BC progression, we focused on transcriptomic changes due to miR-24-2-5p overexpression. Some human genes, such as GNAI3 [15], KLF4 and C-MYC [37], and PKC-alpha [12], have already been described as direct targets of miR-24-2-5p, playing a role in osteogenic differentiation, stemness properties of embryonic stem cells, and survival of BC cells, respectively. Here, we used a high-throughput method, the RNA-seq, to specifically evaluate transcripts modulated in NW1 and MCF7 cells after acute overexpression of miR-24-2-5p. Most of the transcripts shown to be dysregulated upon miR-24-2-5p overexpression were actually downregulated (Fig. 3), being in line with the canonical effect of miRNAs as negative gene expression regulators. Moreover, 30 transcripts were in common between NW1 and MCF7 cells, suggesting that miR-24-2-5p modulates tumour cell functions through similar molecular pathways irrespective of the BC subtypes. A number of transcripts (CNNM4, DCTD, FMR1, PIGS, HLA-A, ICK, SH3BGRL2, WDFY, TRAF9B, IL6ST, PEX10, TRIM59) were further validated by real-time qPCR to be downregulated by miR-24-2-5p overexpression in comparison to control at an early and/or late timepoint post-transfection (Fig. 4). Among these transcripts, WDFY1 and SH3BGRL2 may be of particular interest. WDFY1 is a regulator of endocytic activity in breast and prostate cancer cells [38, 39], and it is also associated with enhanced tumour growth and lung metastasis in a mouse model of BC [39]. SH3BGRL12 expression has been reported to enhance migratory and invasive properties of BC cells in vitro, and their capacity to form lung metastasis in vivo [40]. Finally, we have found relatively poor correlations between a small group of miR-24-2-5p-modulated transcripts (IL6ST, PEX10, CNNM4) and miR-24-2-5p expression levels in BC tissues from patients, suggesting that the gene expression of these transcripts at the primary site is likely subjected to the regulation of other regulatory pathways. Thus, our results remained merely indicative of the role of miR-24-2-5p in primary BC tissues.

We then chose to examine the effect of miR-24-2-5p overexpression in BC cells at a very early stage of bone metastasis formation (i.e., 9 days post tumour cell inoculation in animals), at which time disseminated BC cells in bone are present only as small cell clusters and start to disrupt normal bone homeostasis. In the clinic, high baseline levels of CTX-I, a marker for bone resorption, and P1NP, a marker for bone formation, in the serum from early-stage breast cancer patients are predictive of future bone recurrence, indicating that there is a relationship between accelerated baseline bone turnover and subsequent distant recurrence events [24]. Thus, bone turnover markers are a powerful hallmark of the presence of disseminated BC cells in bone at an early stage, while other techniques (e.g., histology of bone sections or flow-cytometry of bone marrow) show reduced sensibility in disseminated cells detection. Here, we measured CTX-I and P1NP serum levels in animals as a complementary method to the use of BLI for detecting disseminated BC cells. We observed that, in addition to a decreased BLI signal, circulating CTX-1 and P1NP levels were significantly lower in mice injected with miR-24-2-5p-overexpressing NW1 cells than those observed in mice injected with control NW1 cells (Fig. 5). These data indicate that miR-24-2-5p had a protective effect by reducing bone metastasis formation and the bone turnover in vivo.

Acknowledging the importance of the cross-talk between cancer cells and bone cells in the formation of BC metastasis in bone [2], and in the light of these results obtained in vivo, we asked whether miR-24-2-5p had any influence on osteoclast differentiation in vitro. Thus, we tested the effect of the conditioned medium (CM) from miR-24-2-5p-overexpressing BC cells during human and murine osteoclastogenesis assays. We found that the CM collected from human BC cells overexpressing miR-24-2-5p inhibited both human and murine osteoclast differentiation (Fig. 6). In addition, we observed that miR-24-2-5p expression levels in osteoclasts cultured with the CM from miR-24-2-5p-overexpressing cells were substantially increased (∼ 180-fold) compared to levels seen in the control group (Fig. 6F), and that there was a ∼ 350-fold increase of miR-24-2-5p expression levels in the CM from miR-24-2-5p-overexpressing NW1 cells compared to the CM from control NW1 cells (Supplementary Fig. 12), indicating a direct relationship between high miR-24-2-5p levels in osteoclasts and those observed in the CM from miR-24-2-5p-overexpressing NW1 cells. As a limitation of our study, we cannot exclude an indirect effect of miR-24-2-5p on osteoclasts on the secretion of other factors by BC cells. However, it is highly likely that tumour-derived miR-24-2-5p directly influenced osteoclast differentiation. This contention is supported by several examples in the literature of tumour-derived miRNAs (e.g., miR-21, miR-141, miR-192, miR-940) that are uptaken by bone cells, acting as negative regulators of gene expression in these recipient cells [2, 10, 25]. Our study is mainly focussed to evaluate the role of miR-24-2-5p derived from BC cells, however the role of miR-24-2-5p in bone will require further investigation.

## Conclusion

MiR-24-2-5p has a protective role in the progression of BC cells to bone. MiR-24-2-5p forced expression decreased proliferative, migratory, and invasive properties of BC cells in vitro, and bone metastasis formation in animals. Furthermore, tumour-derived miR-24-2-5p reduced BC-induced osteoclast differentiation in vitro, and BC-induced bone turnover in vivo, which likely contributed to the reduction of BC cell dissemination in animals. Overall, miR-24-2-5p act as a tumour suppressor miRNA in the early stages of breast-to-bone metastasis.

## Electronic supplementary material

Below is the link to the electronic supplementary material.

Supplementary Material 1

Supplementary Material 2: List of primers used in real-time qPCR, and ‘miRBAse’ accession numbers (https://mirbase.org, Accessed on May 2021) of miR-24-2 forms.

Supplementary Material 3: List of predicted targets (TargetScan) for miR-24-2-5p.

Supplementary Material 4: GSEA analysis on genes modulated by miR-24-2-5p overexpression in NW1 and MCF7 cells and detected by RNA-seq.

Supplementary Material 5: ClueGo-based analysis on the top 250 predicted targets of miR-24-2-5p.

## Data Availability

All data presented in this work are present in the paper and/or in the Supplementary Materials. RNA-seq data are available on the Gene Expression Omnibus (GEO) dataset, NCBI (Accession: GSE267064). Other data that support the findings of this study are available from corresponding authors (MP, PC) on reasonable request. The dataset used or analysed during the current study are available from corresponding authors (MP, PC) on reasonable request.

## References

[1] Parker AL, Benguigui M, Fornetti J, Goddard E, Lucotti S, Insua-Rodríguez J, et al. Current challenges in metastasis research and future innovation for clinical translation. Clin Exp Metastasis. 2022;39(2):263–77. 35072851 10.1007/s10585-021-10144-5 PMC8971179

[2] Clézardin P, Coleman R, Puppo M, Ottewell P, Bonnelye E, Paycha F, et al. Bone metastasis: mechanisms, therapies, and biomarkers. Physiol Rev. 2021;101(3):797–855. 33356915 10.1152/physrev.00012.2019

[3] Dafni U, Tsourti Z, Alatsathianos I. Breast Cancer statistics in the European Union: incidence and survival across European Countries. Breast Care (Basel). 2019;14(6):344–53. 31933579 10.1159/000503219 PMC6940474

[4] Ye F, Dewanjee S, Li Y, Jha NK, Chen ZS, Kumar A, et al. Advancements in clinical aspects of targeted therapy and immunotherapy in breast cancer. Mol Cancer. 2023;22(1):105. 37415164 10.1186/s12943-023-01805-y PMC10324146

[5] , *et al.* 3rd ESO–ESMO International Consensus Guidelines for Advanced Breast Cancer(ABC 3). Annals of Oncology. 2017;28(1):16–33. 10.1093/annonc/mdw544 PMC5378224 28177437

[6] Valachis A, Carlqvist P, Ma Y, Szilcz M, Freilich J, Vertuani S, et al. Overall survival of patients with metastatic breast cancer in Sweden: a nationwide study. Br J Cancer. 2022;127(4):720–5. 35597870 10.1038/s41416-022-01845-z PMC9381497

[7] Ha M, Kim VN. Regulation of microRNA biogenesis. Nat Rev Mol Cell Biol. 2014;15(8):509–24. 25027649 10.1038/nrm3838

[8] Puppo M, Valluru MK, Clézardin P. Chapter 33 - MicroRNAs and bone metastasis: how small RNAs regulate secondary tumor formation and progression in the skeleton. In: Heymann D, editor. Bone sarcomas and bone metastases - from bench to Bedside. Third Edition): Academic; 2022. pp. 457–69.

[9] Puppo M, Valluru MK, Clézardin P. MicroRNAs and their roles in breast Cancer bone metastasis. Curr Osteoporos Rep. 2021;19(3):256–63. 33830428 10.1007/s11914-021-00677-9 PMC8310490

[10] Puppo M, Valluru MK, Croset M, Ceresa D, Iuliani M, Khan A et al. MiR-662 is associated with metastatic relapse in early-stage breast cancer and promotes metastasis by stimulating cancer cell stemness. Br J Cancer. 2023;754–71. 10.1038/s41416-023-02340-9 PMC10449914 37443350

[11] Wang S, Liu N, Tang Q, Sheng H, Long S, Wu W. MicroRNA-24 in Cancer: a double side medal with Opposite Properties. Front Oncol. 2020;10:553714. 33123467 10.3389/fonc.2020.553714 PMC7566899

[12] Martin EC, Elliott S, Rhodes LV, Antoon JW, Fewell C, Zhu Y, et al. Preferential star strand biogenesis of pre-mir-24-2 targets PKC-alpha and suppresses cell survival in MCF-7 breast cancer cells. Mol Carcinog. 2014;53(1):38–48. 22911661 10.1002/mc.21946 PMC4030540

[13] Hassan MQ, Gordon JA, Beloti MM, Croce CM, van Wijnen AJ, Stein JL, et al. A network connecting Runx2, SATB2, and the miR-23a ∼ 27a ∼ 24 – 2 cluster regulates the osteoblast differentiation program. Proc Natl Acad Sci U S A. 2010;107(46):19879–84. 20980664 10.1073/pnas.1007698107 PMC2993380

[14] Zeng H-C, Bae Y, Dawson BC, Chen Y, Bertin T, Munivez E, et al. MicroRNA miR-23a cluster promotes osteocyte differentiation by regulating TGF-β signalling in osteoblasts. Nat Commun. 2017;8(1):15000. 28397831 10.1038/ncomms15000 PMC5394267

[15] Meng L, Yuan L, Ni J, Fang M, Guo S, Cai H, et al. Mir24-2-5p suppresses the osteogenic differentiation with Gnai3 inhibition presenting a direct target via inactivating JNK-p38 MAPK signaling axis. Int J Biol Sci. 2021;17(15):4238–53. 34803495 10.7150/ijbs.60536 PMC8579458

[16] Wang N, Reeves KJ, Brown HK, Fowles ACM, Docherty FE, Ottewell PD, et al. The frequency of osteolytic bone metastasis is determined by conditions of the soil, not the number of seeds; evidence from in vivo models of breast and prostate cancer. J Experimental Clin Cancer Res. 2015;34(1):124. 10.1186/s13046-015-0240-8 PMC4615337 26480944

[17] Croset M, Pantano F, Kan CWS, Bonnelye E, Descotes F, Alix-Panabières C, et al. miRNA-30 family members inhibit breast Cancer Invasion, Osteomimicry, and Bone Destruction by directly targeting multiple bone metastasis-Associated genes. Cancer Res. 2018;78(18):5259–73. 30042152 10.1158/0008-5472.CAN-17-3058

[18] Bindea G, Galon J, Mlecnik B. CluePedia Cytoscape plugin: pathway insights using integrated experimental and in silico data. Bioinformatics. 2013;29(5):661–3. 23325622 10.1093/bioinformatics/btt019 PMC3582273

[19] Canuas-Landero VG, George CN, Lefley DV, Corness H, Muthana M, Wilson C, et al. Oestradiol contributes to Differential Antitumour effects of Adjuvant Zoledronic Acid observed between pre- and post-menopausal women. Front Endocrinol (Lausanne). 2021;12:749428. 34733240 10.3389/fendo.2021.749428 PMC8559775

[20] Allocca G, Hughes R, Wang N, Brown HK, Ottewell PD, Brown NJ, et al. The bone metastasis niche in breast cancer: potential overlap with the haematopoietic stem cell niche in vivo. J bone Oncol. 2019;17:100244. 31236323 10.1016/j.jbo.2019.100244 PMC6582079

[21] Couch Y, Buzàs EI, Di Vizio D, Gho YS, Harrison P, Hill AF, et al. A brief history of nearly EV-erything - the rise and rise of extracellular vesicles. J Extracell Vesicles. 2021;10(14):e12144. 34919343 10.1002/jev2.12144 PMC8681215

[22] Welsh J. Chapter 40 - animal models for studying Prevention and treatment of breast Cancer. In: Conn PM, editor. Animal models for the study of Human Disease. Boston: Academic; 2013. pp. 997–1018.

[23] Mukherjee S, Shelar B, Krishna S. Versatile role of miR-24/24 – 1*/24 – 2* expression in cancer and other human diseases. Am J Transl Res. 2022;14(1):20–54. 35173828 PMC8829624

[24] Brown J, Rathbone E, Hinsley S, Gregory W, Gossiel F, Marshall H, et al. Associations between serum bone biomarkers in early breast Cancer and development of bone metastasis: results from the AZURE (BIG01/04) trial. JNCI: J Natl Cancer Inst. 2018;110(8):871–9. 29425304 10.1093/jnci/djx280 PMC6093369

[25] Puppo M, Jaafar M, Diaz JJ, Marcel V, Clézardin P. MiRNAs and snoRNAs in bone metastasis: functional roles and clinical potential. Cancers (Basel). 2022;15(1). 10.3390/cancers15010242 PMC9818347 36612237

[26] Hong Sung K, Na Kyung L. Gene expression profiling in osteoclast precursors by insulin using microarray analysis. Mol Cells. 2014;37(11):827–32. 25377254 10.14348/molcells.2014.0223 PMC4255103

[27] Sanpaolo ER, Rotondo C, Cici D, Corrado A, Cantatore FP. JAK/STAT pathway and molecular mechanism in bone remodeling. Mol Biol Rep. 2020;47(11):9087–96. 33099760 10.1007/s11033-020-05910-9 PMC7674338

[28] Min Y, Ren X, Vaught DB, Chen J, Donnelly E, Lynch CC, et al. Tie2 signaling regulates osteoclastogenesis and osteolytic bone invasion of breast cancer. Cancer Res. 2010;70(7):2819–28. 20233869 10.1158/0008-5472.CAN-09-1915 PMC2848896

[29] Kang R, Zeh HJ, Lotze MT, Tang D. The beclin 1 network regulates autophagy and apoptosis. Cell Death Differ. 2011;18(4):571–80. 21311563 10.1038/cdd.2010.191 PMC3131912

[30] Boissy P, Saltel F, Bouniol C, Jurdic P, Machuca-Gayet I. Transcriptional activity of nuclei in multinucleated osteoclasts and its modulation by calcitonin. Endocrinology. 2002;143(5):1913–21. 11956174 10.1210/endo.143.5.8813

[31] Wang Z. The guideline of the design and validation of MiRNA mimics. Methods Mol Biol. 2011;676:211–23. 20931400 10.1007/978-1-60761-863-8_15

[32] Hong DS, Kang YK, Borad M, Sachdev J, Ejadi S, Lim HY, et al. Phase 1 study of MRX34, a liposomal miR-34a mimic, in patients with advanced solid tumours. Br J Cancer. 2020;122(11):1630–7. 32238921 10.1038/s41416-020-0802-1 PMC7251107

[33] Yang X, Wang L, Li R, Zhao Y, Gu Y, Liu S, et al. The long non-coding RNA PCSEAT exhibits an oncogenic property in prostate cancer and functions as a competing endogenous RNA that associates with EZH2. Biochem Biophys Res Commun. 2018;502(2):262–8. 29803673 10.1016/j.bbrc.2018.05.157

[34] Croset M, Goehrig D, Frackowiak A, Bonnelye E, Ansieau S, Puisieux A, et al. TWIST1 expression in breast Cancer cells facilitates bone metastasis formation. J Bone Miner Res. 2014;29(8):1886–99. 24619707 10.1002/jbmr.2215

[35] Ahmad N, Kushwaha P, Karvande A, Tripathi AK, Kothari P, Adhikary S, et al. MicroRNA-672-5p identified during weaning reverses Osteopenia and Sarcopenia in Ovariectomized Mice. Mol Ther Nucleic Acids. 2019;14:536–49. 30769134 10.1016/j.omtn.2019.01.002 PMC6374523

[36] Lee SWL, Paoletti C, Campisi M, Osaki T, Adriani G, Kamm RD, et al. MicroRNA delivery through nanoparticles. J Controlled Release. 2019;313:80–95. 10.1016/j.jconrel.2019.10.007 PMC6900258 31622695

[37] Lee SH, Chen TY, Dhar SS, Gu B, Chen K, Kim YZ, et al. A feedback loop comprising PRMT7 and mir-24-2 interplays with Oct4, nanog, Klf4 and c-Myc to regulate stemness. Nucleic Acids Res. 2016;44(22):10603–18. 27625395 10.1093/nar/gkw788 PMC5159542

[38] Dutta S, Roy S, Polavaram NS, Stanton MJ, Zhang H, Bhola T, et al. Neuropilin-2 regulates endosome maturation and EGFR trafficking to Support Cancer Cell Pathobiology. Cancer Res. 2016;76(2):418–28. 26560516 10.1158/0008-5472.CAN-15-1488 PMC4715955

[39] Kesavan R, Frömel T, Zukunft S, Laban H, Geyer A, Naeem Z, et al. Cyp2c44 regulates prostaglandin synthesis, lymphangiogenesis, and metastasis in a mouse model of breast cancer. Proc Natl Acad Sci U S A. 2020;117(11):5923–30. 32123095 10.1073/pnas.1921381117 PMC7084120

[40] Li DD, Deng L, Hu SY, Zhang FL, Li DQ. SH3BGRL2 exerts a dual function in breast cancer growth and metastasis and is regulated by TGF-β1. Am J Cancer Res. 2020;10(4):1238–54. 32368399 PMC7191107

