# Supplementary material for: Protective effects of miR-24-2-5p in early stages of breast cancer bone metastasis
Source: Breast Cancer Res. 2024 Dec 18;26:186. doi: 10.1186/s13058-024-01934-2 (PMC11656574; doi:10.1186/s13058-024-01934-2)
Supplement: Supplementary file 1 — Supplementary Material 1 [file 13058_2024_1934_MOESM1_ESM.docx]

**Supplementary figures.**

**Figure S1 – Clinical details on BC patients’ cohort.** Early-stage BC patients (N=48) belonging to a larger cohort (AZURE) have been selected for this study, and divided into 3 groups (NOMET, BONEMET, SOFTMET) based on metastatic outcomes. Clinico-biological characteristics of BC patients are here displayed alongside relative percentages.

**
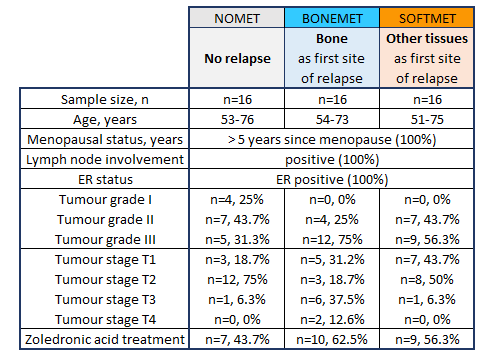
**

**Figure S2 –** Recurrence-free probability in bone for high or low levels of circulating miR-24-2-5p levels in early-stage BC patients from our cohort (N=48).

**
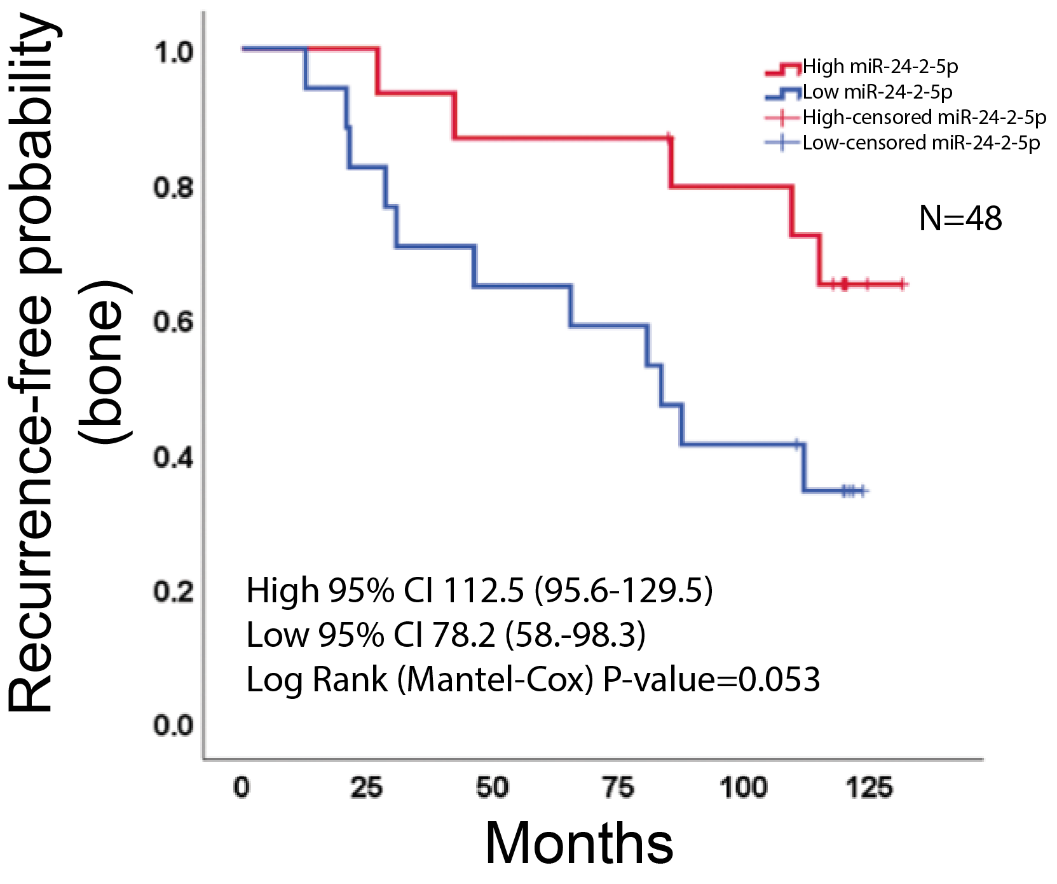
**

**Figure S3 – Raw cycle threshold (CT) values and expression of miR-24 forms in serum of early-stage BC patients. A)** Raw CT value detection by TLDA of miR-24-3p, miR-24-1-5p and miR-24-2-5p in Azure patients’ cohort. MiR-24-1-5p and miR-24-2-5p CT values are higher compared to miR-24-3p, the latter being the common form between chromosome 9 and 19. **B)** Expression of miR-24-3p, miR-24-1-5p and miR-24-2-5p after global mean normalization (GMN). MiR-24-1-5p and miR-24-2-5p expressions are lower compared to miR-24-3p. Student t-test, p-value<0.0001.


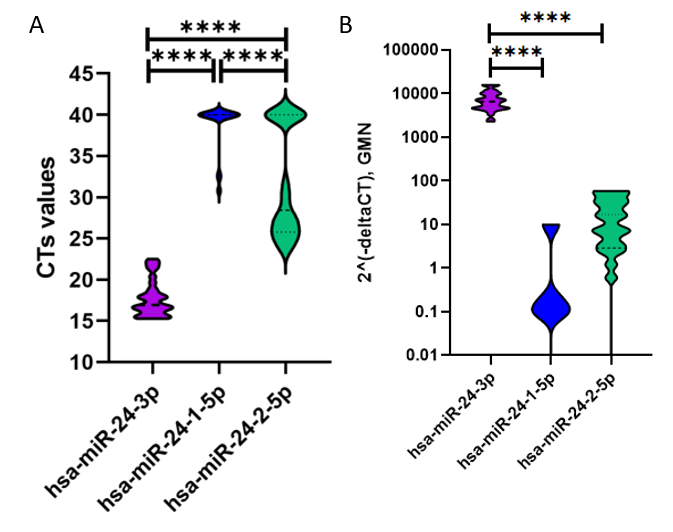


**Figure S4 – MiR-24-2-5p overexpression in BC cell lines. A)** Relative miR-24-2-5 expression levels in NW1 cells transfected with miRNA mimics for miR-24-2-5p (MIMIC-miR-24-2-5p) or negative control miRNA mimics (MIMIC-negCtrl). Cell pellet was collected at 1 day and 4 days post-transfection. **B)** Relative miR-24-2-5 expression levels in engineered NW1 cells (NW1/miR-negCtrl; NW1/miR-24-2-5p). **C)** Relative miR-24-2-5 expression levels in MCF7 cells transfected with miRNA mimics for miR-24-2-5p (MIMIC-miR-24-2-5p) or negative control miRNA mimics (MIMIC-negCtrl). Cell pellets were collected at 1 day and 3 days post-transfection. **D)** Relative miR-24-2-5p expression levels in engineered MCF7 cells (MCF7/miR-negCtrl; MCF7/miR-24-2-5p). For all panels, relative gene expression of miR-24-2-5p was obtained by comparing miR-24-2-5p-overexpressing cells to relative control cells and calculated by 2^-ΔΔCT^ method. U6 has been used as a housekeeping gene. Means of two independent experiments ± SEM were shown for all experiments. * p≤0.05, ** p<0.01, *** p<0.001.


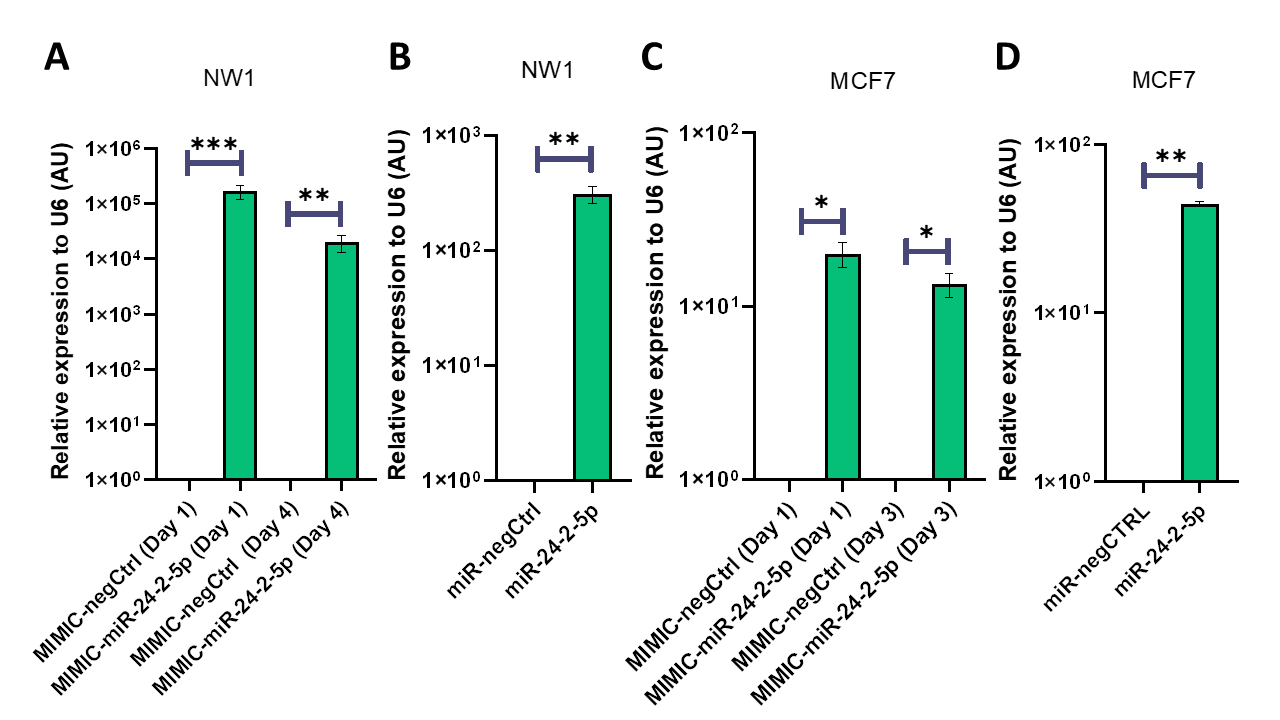


**Figure S5 – Inhibition of miR-24-2-5p expression.** Relative miR-24-2-5p expression levels in human BC cell lines (ZR-75-1, T-47D, SK-BR-3) transfected with miR-24-2-5p inhibitor (INHIB-miR-24-2-5p) compared to their control counterparts transfected with a negative control inhibitor (INHIB-negCTRL) at 24 hours post-transfection. For all graphs, relative gene expression levels of miR-24-2-5p was calculated by 2-ΔΔCT method, and U6 was used as a housekeeping gene. Data are the mean ± SEM of two independent experiments, * p≤0.05, ** p<0.01, **** p<0.0001*.*

***
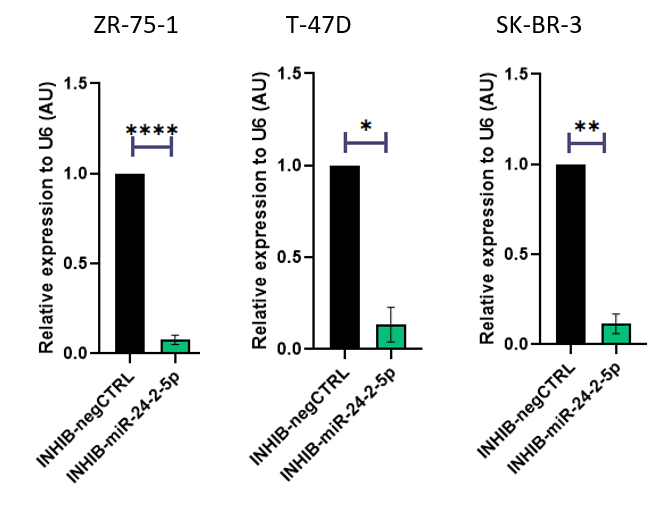
***

**Figure S6 – Proliferation assay.** Proliferation assay with human ZR-75-1, T-47D, and SK-BR-3 cells transfected with INHIB-miR-24-2-5p or negative control (INHIB-negCTRL) performed for six consecutive days post-transfection (from day 0 to day 5). Proliferation rates were normalised to day 0. Data are the mean ± SEM of 2 independent experiments.

**
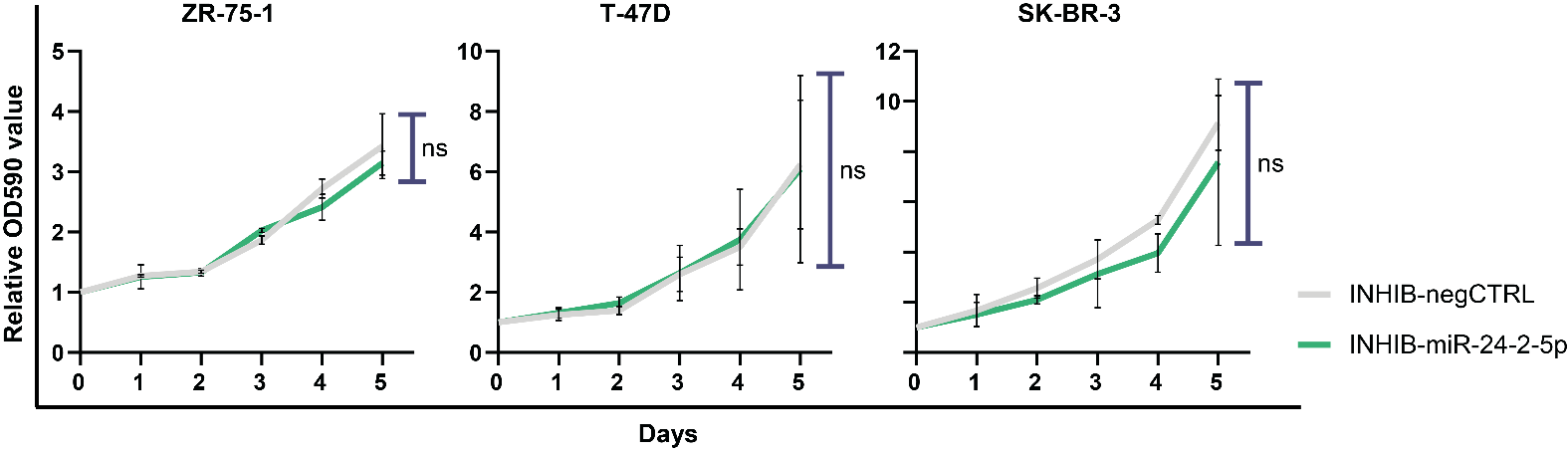
**

**Figure S7 – Migration assay.** 48h-cell migration assay of human ZR-75-1, T-47D, and SK-BR-3 breast cancer cells transfected with a miR-24-2-5p inhibitor (INHIB-miR-24-2-5p), compared to their control counterparts transfected with a negative control inhibitor (INHIB-negCTRL). Data are the mean ± SEM of two independent experiments.

**
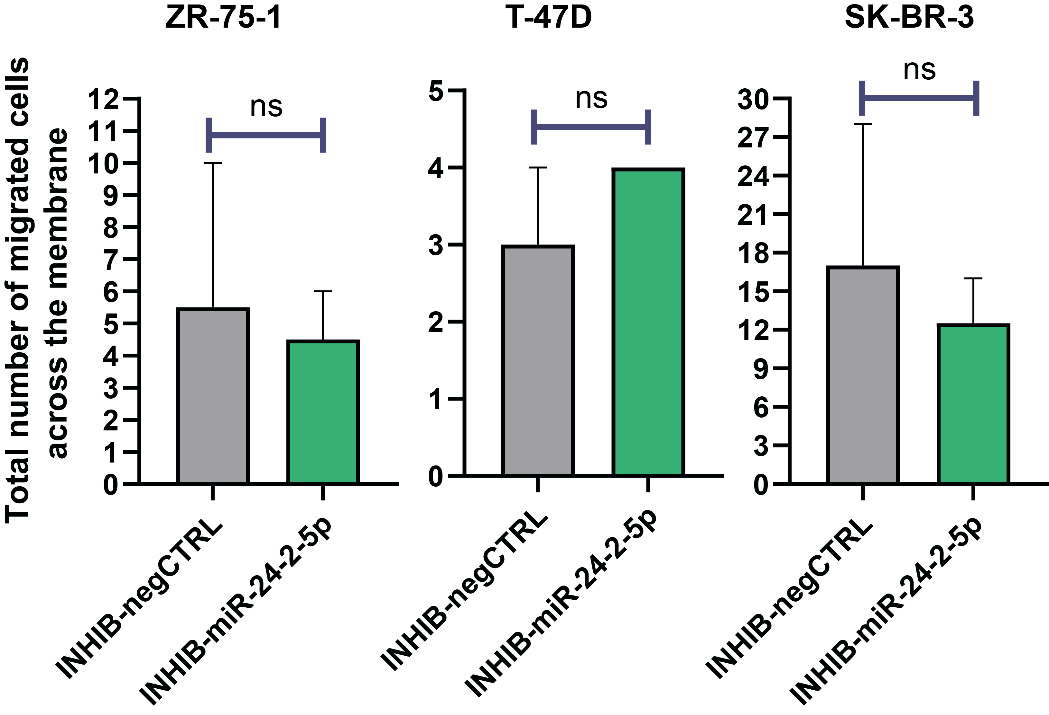
**

**Figure S8 – Real-time qPCR validation of miR-24-2-5p mRNA targets.** Expression of transcripts that resulted downregulated in the RNA-seq data upon miR-24-2-5p overexpression. MINPP1 and ZNF770 were not downregulated at any timepoint (D1,4) in NW1 cells transiently transfected with MIMIC-miR-24-2-5p *versus* relative negative MIMIC control (MIMIC-negCTRL).

**Figure S9 – Measurement of circulating CTX-I levels in mice.** Circulating CTX-I levels measured in serum from naïve mice (dark grey, NAIVE) at the same age of mice belonging to the control (grey, MIMIC-negCTRL) and experimental (green, MIMIC-miR-24-2-5p) groups used in the present study.

**Figure S10 – ClueGO-based analysis.** ClueGO-based analysis conducted on the top 250 predicted targets (TargetScan) for miR-24-2-5p. GO biological process networks and their interactions are here represented as s network map.


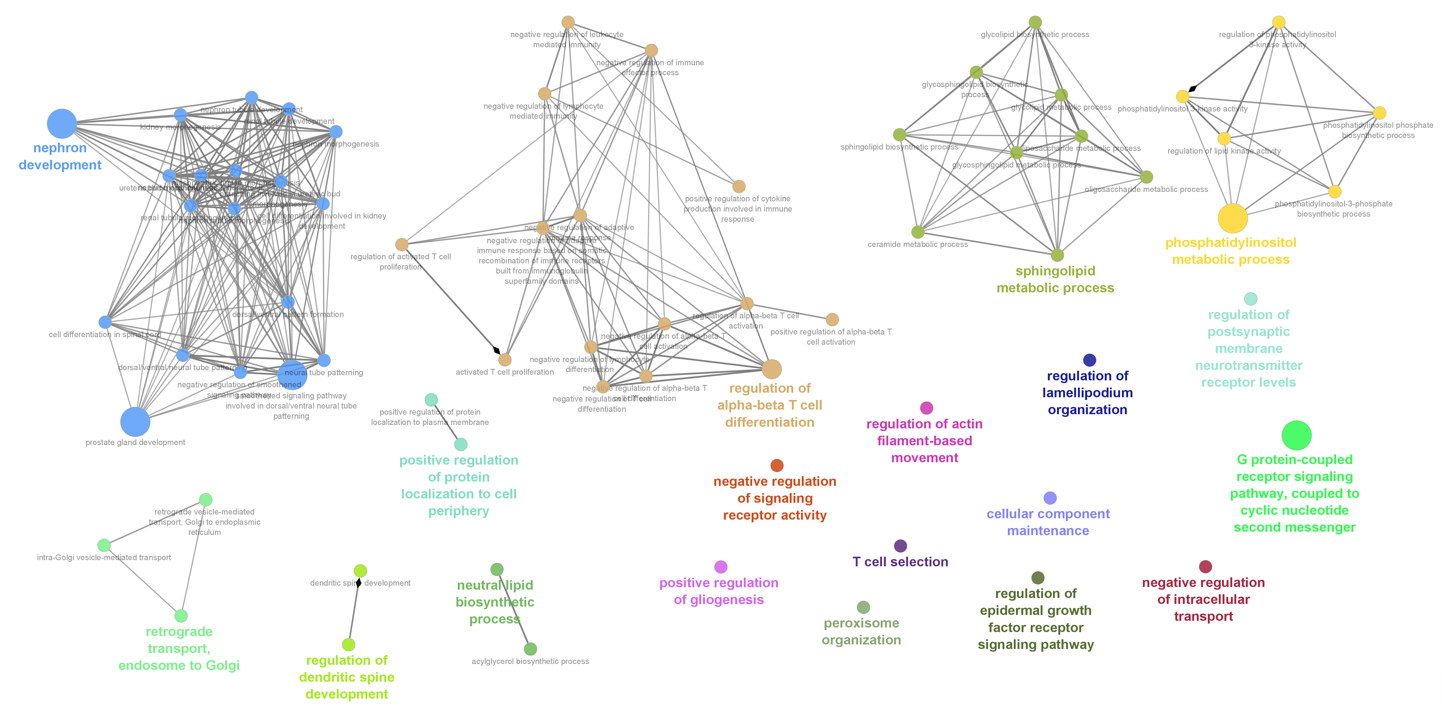


**Figure S11 – Osteoclastogenesis assays**. Representative figures of wells containing differentiated osteoclasts after 12 days in culture in the presence of CM media from control (MIMIC-negCTRL) or miR-24-2-5p overexpressing (MIMIC-miR-24-2-5p) NW1 or MCF-7 cells (upper and lower panels, respectively). Scale bars: 100µM.


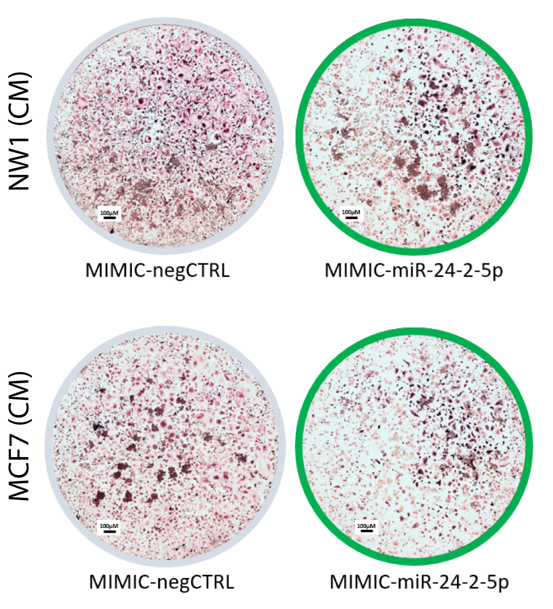


**Figure S12 – Real-time qPCR validation for key genes associated with osteoclastogenesis**. Gene expression analysis on human osteoclasts treated with the conditioned medium (CM) from NW1 or MCF-7 breast cancer cells overexpressing miR-24-2-5p (MIMIC-miR-24-2-5p) or a control mimic (MIMIC-negCTRL). Relative expression levels of ACP5 (encoding for TRAP), CTSK, MMP-9, and CALCR genes compared to RPL32 as housekeeping gene were measured by real-time qPCR and calculated by 2-ΔΔCT method. Data are the mean ± SEM of 3 independent experiments. * p≤0.05, ** p<0.01.


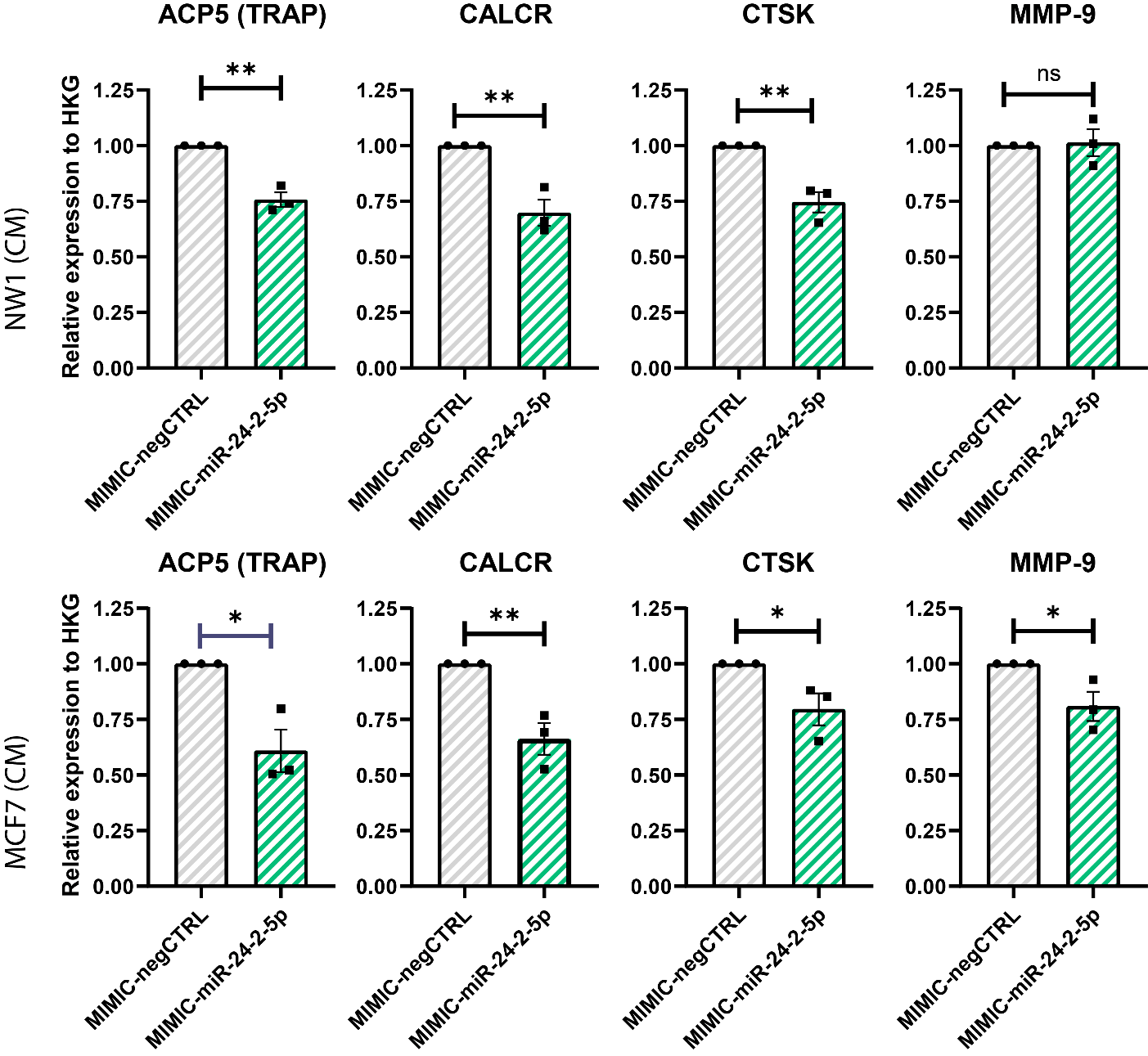


**Figure S13 –** Quantification of miR-24-2-5p expression levels in the CM collected from miR-24-2-5p-overexpressing (MIMIC/miR-24-2-5p) or control (MIMIC-negCTRL) NW1 cells, and then used for osteoclastogenesis assays. Data are the mean ± SEM of independent experiments. ** p<0.01.

**Figure S14 – Osteoclastogenesis.** Representative images of immature and mature TRAP-positive murine bone marrow-derived osteoclasts at different time of their differentiation. 2.5X magnification.


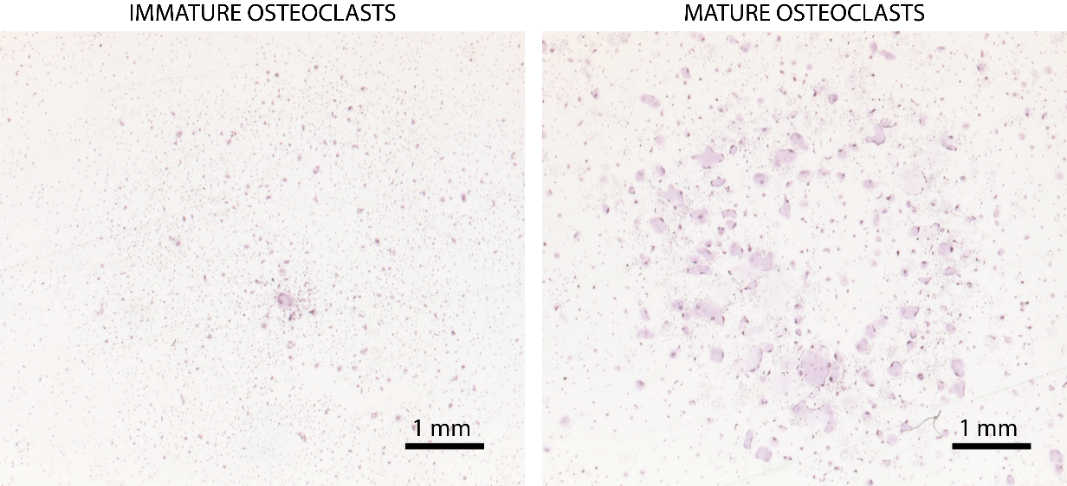


**Figure S15 - Gene expression analysis of immature and mature murine osteoclasts**. Relative expression levels of Atp6vab2, Acp5, Traf6, Tnfrsf11a, Nfkb1, Mmp9, Mitf, Itgb3, Ctsk, Car2, and Calcr genes compared to RPL32 as housekeeping gene were calculated by 2-ΔΔCT method. Data are the mean ± SEM of 3 independent experiments, * p≤0.05, ** p<0.01, *** p<0.001, **** p<0.0001.

*
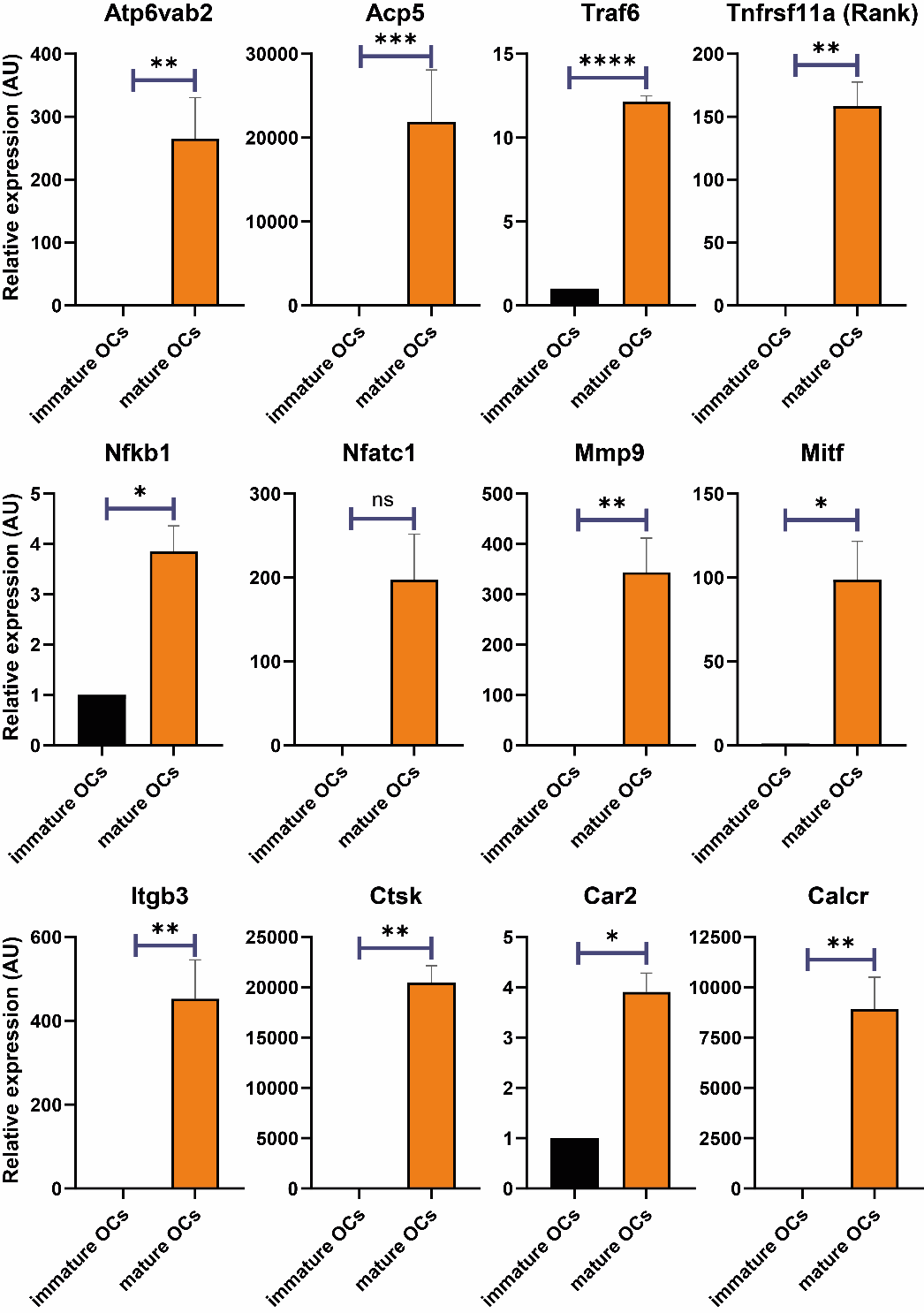
*
